# Testing the Complete Plastome for Species Discrimination, Cryptic Species Discovery and Phylogenetic Resolution in *Cephalotaxus* (Cephalotaxaceae)

**DOI:** 10.3389/fpls.2022.768810

**Published:** 2022-05-04

**Authors:** Jie Wang, Chao-Nan Fu, Zhi-Qiong Mo, Michael Möller, Jun-Bo Yang, Zhi-Rong Zhang, De-Zhu Li, Lian-Ming Gao

**Affiliations:** ^1^CAS Key Laboratory for Plant Diversity and Biogeography of East Asia, Kunming Institute of Botany, Chinese Academy of Sciences, Kunming, China; ^2^Germplasm Bank of Wild Species, Kunming Institute of Botany, Chinese Academy of Sciences, Kunming, China; ^3^College of Life Sciences, University of Chinese Academy of Sciences, Beijing, China; ^4^Royal Botanic Garden Edinburgh, Edinburgh, United Kingdom; ^5^Lijiang Forest Biodiversity National Observation and Research Station, Kunming Institute of Botany, Chinese Academy of Sciences, Lijiang, China

**Keywords:** *Cephalotaxus*, complete plastome, species discrimination, cryptic species, phylogenetic relationships, standard DNA barcodes

## Abstract

Species of *Cephalotaxus* have great economic and ecological values. However, the taxonomy and interspecific phylogenetic relationships within the genus have been controversial and remained not fully resolved until now. To date, no study examined the efficiency of the complete plastome as super-barcode across *Cephalotaxus* species with multiple samples per taxon. In this study, we have evaluated the complete plastome in species discrimination and phylogenetic resolution in *Cephalotaxus* by including 32 individuals of all eight recognized species and five varieties following Farjon’s classification (2010) with multiple samples per taxon. Our results indicated that not all species recognized in recent taxonomic revisions of *Cephalotaxus* could be distinguished and not all were monophyletic. Based on the plastome phylogeny, a new taxonomic classification for the genus comprising nine species and two varieties, including a cryptic species, was proposed. The phylogeny also resolved all interspecific relationships. Compared to the plastome based classification, standard DNA barcodes, alone or in combination, only recognized a maximum of seven out of the nine species. Moreover, two highly variable single loci, *ycf*1 and *rps*16, each alone achieved full species discrimination. With the moderate length of 1079 bp, *rps*16 is proposed as a specific barcode to discriminate *Cephalotaxus* species. The super-barcodes and specific barcode candidates will aid in the identification of endangered *Cephalotaxus* species, and to help focus conservation measures.

## Introduction

Species delimitation is fundamental in many areas of biology (Duminil and Di Michele, 2009). Traditional species delimitation is based on the analysis of morphological variation, which is, however, affected by a large number of factors, and can result in difficulties in species identification (Hebert et al., 2003). With the development of molecular phylogenetic methods, applications of the “phylogenetic species concept” have increased, which uses monophyly to define species (de Queiroz, 1998, 2005; Zimmers et al., 2017). DNA barcoding is a technique to identify biological species, parts and products using one or several standardized DNA regions (Hebert et al., 2003; Kress et al., 2005; Hollingsworth, 2011; Hollingsworth et al., 2016). It has been widely used as a molecular tag for species identification and delimitation, and is contributing to rapid scientific progress on diverse fronts (Joly et al., 2014; Kress et al., 2015; Hollingsworth et al., 2016). Although a number of DNA markers were assessed as candidate DNA barcodes for plants, no barcode alone performed as well as *COI* in animals (Hollingsworth, 2011). Several different combinations of candidate DNA regions were proposed for barcoding plants (Chase et al., 2007; Kress and Erickson, 2007; Pennisi, 2007; Hollingsworth et al., 2009). However, the standard DNA barcodes suggested for flowering plants (*rbc*L, *mat*K, *trn*L-*trn*F, and *trn*H-*psb*A) generally do not perform well across closely related species and recently diverged species mainly due to the lack of adequate genetic variation (Hollingsworth et al., 2009; Li et al., 2011; Yan et al., 2015; Coissac et al., 2016).

With the rapid development of next-generation sequencing (NGS) technologies and decreasing sequencing costs, a large amount of genomic data can be rapidly generated to date, thus the concept of DNA barcodes has been expanded (Coissac et al., 2016; Hollingsworth et al., 2016; Tonti-Filippini et al., 2017). The complete plastome was proposed as “ultra-barcode” (Nock et al., 2011; Kane et al., 2012) or “super-barcode” (Yang et al., 2013) for plant species delimitation, being referred to as next-generation DNA barcode (Hollingsworth et al., 2016). Compared to standard DNA barcodes, next-generation DNA barcodes may overcome the inherent limitations of traditional DNA barcodes, as they potentially possess more informative and variable sites, and therefore can enhance species discrimination power and phylogenetic resolution (Kane et al., 2012; Hollingsworth et al., 2016; Ji et al., 2019). Genome skimming is a cost-effective NGS technology to obtain the whole plastid genome (Straub et al., 2012; Ripma et al., 2014). The discriminatory efficiency of the complete plastome in plants has been assessed in several recent studies (e.g., Turner et al., 2016; Fu et al., 2019; Ji et al., 2019; Song et al., 2020). However, very few studies have been undertaken using multiple individuals per species from multiple congeneric species.

The plastid genome is uniparentally inherited in most plants (Birky, 2001; Gitzendanner et al., 2018) with an exception for most conifers such as Cephalotaxaceae, Taxaceae and Pinaceae where it is paternally inherited (Gianordoli, 1974; Mogensen, 1996), and behaves as a single non-recombining locus, providing a strong signal of phylogenetic history (Petit and Vendramin, 2007; Yu et al., 2018). With a highly conserved gene order and absence of recombination, the plastid genome is an ideal target for comparative analysis across land plants (Wicke et al., 2011; Hollingsworth et al., 2016; Gitzendanner et al., 2018). The benefits of the complete plastome have been argued for its utilization in plant species delimitation, although this may not solve delimitation failures resulting from rare biparental plastid inheritance, introgression and hybridization, such as presumed for *Salix* (Percy et al., 2014), and *Calligonum* (Song et al., 2020). However, plastome barcoding has shown a great promise for reliably distinguishing closely related species (Kane et al., 2012; Ruhsam et al., 2015; Zhang et al., 2017; Zhu et al., 2018; Fu et al., 2022). In addition, the plastid genome has a large number of evolutionarily informative variation, which has been widely used for phylogenetic reconstruction at deep to shallow levels in land plants (Moore et al., 2010; Yu et al., 2018; Ji et al., 2019; Li et al., 2019, 2021), including conifers (e.g., Fu et al., 2019; Zhang et al., 2019; Ji et al., 2021).

The genus *Cephalotaxus* Siebold and Zucc. ex Endl. is a small group of 7-10 species of evergreen conifers belonging to family Cephalotaxaceae in a narrow sense (Singh, 1961; Farjon, 1998, 2010) or Taxaceae in a wider sense (Hart, 1987; Price, 1990, 2003; Christenhusz et al., 2011). The plants in this genus are understory trees distributed in eastern Asia, north of the Indo-Chinese peninsula and Himalayas, mainly in China, Korea, Japan, Vietnam, Laos, Thailand, India, and Myanmar (Fu et al., 1999; Farjon, 2010; Lang et al., 2013; Yang et al., 2017; Zhang et al., 2019). The taxonomy of *Cephalotaxus* is primarily based on morphological characters and geographical distribution of individual taxa (Farjon, 2010; Lang et al., 2011, 2013; Yang et al., 2017). However, the morphological characters used for species delimitation are quite variable and characteristics usually overlap to a high degree between species resulting in a complex and controversial taxonomic history (Farjon, 2010; Lang et al., 2011, 2013; Zhang et al., 2019). For example, Farjon (2010) recognized eight species and three varieties in *Cephalotaxus*, i.e., *C. fortunei* Hook. (including *C. f*. var. *fortunei* and *C. f*. var. *alpina* H.L. Li), *C. hainanensis* H.L. Li., *C. harringtonii* (Knight ex J. Forbes) K. Koch (including *C. h.* var. *harringtonii*, *C. h.* var. *nana* (Nakai) Rehder and *C. h.* var. *wilsoniana* (Hayata) Kitam.), *C. lanceolata* K.M. Feng, *C. latifolia* L.K. Fu and R.R. Mill, *C. mannii* Hook.f., *C. oliveri* Mast., and *C. sinensis* (Rehd. and E.H. Wilson) H.L. Li. Out of these, six species were recognized in China by Fu et al. (1999), but had different taxonomic treatments. For instance, *C. hainanensis* was treated as a synonym of *C. mannii*, and *C. harringtonii* var. *wilsoniana* was treated as a variety of *C. sinensis* (Table 1). Whereas Lang et al. (2013), Zhang et al. (2019) only recognized seven species in recent revisions of the genus but with taxonomic differences (Table 1). Thus, the taxonomy of *Cephalotaxus* is controversial and has not been resolved satisfactorily.

**TABLE 1:** Comparison of different taxonomic treatments of the genus *Cephalotaxus*.

Farjon, 2010	Fu et al., 1999	Lang et al., 2013	Zhang et al., 2019	This paper
8 species	6 species in China	7 species	7 species	9 species
***C. fortunei***	***C. fortunei***			
*C. fortunei* var. *fortunei*	*C. fortunei* var. *fortunei*	***C. fortunei***	***C. fortunei***	***C. fortunei***
*C. fortunei* var. *alpina*	*C. fortunei* var. *alpina*	***C. alpina***	***C. alpina***	***C. alpina***
***C. hainanensis***	(*C. mannii*)	***C. hainanensis***	***C. hainanensis***	***C. hainanensis***
***C. harringtonii***				***C. harringtonii***
*C. harringtonii* var. *harringtonii*		***C. harringtonii***	***C. harringtonii***	*C. harringtonii* var. *harringtonii*
*C. harringtonii* var. *nana*		***C. nana***	***C. nana***	*C. harringtonii* var. *nana*
*C. harringtonii* var. *wilsoniana*	*C. sinensis* var. *wilsoniana*	(*C. harringtonii*)	(*C. harringtonii*)	*C. harringtonii* var. *wilsoniana*
***C. lanceolata***	***C. lanceolata***	(*C. griffithii*)	(*C. griffithii*)	***C. lanceolata***
(*C. mannii*)	(*C. mannii*)	***C. griffithii***	***C. griffithii***	(*C. mannii*)
***C. latifolia***	***C. latifolia***	(*C. nana*)	(*C. nana*)	(*C. sinensis*)
***C. mannii***	***C. mannii***	(*C. harringtonii*)	(*C. hainanensis*)	***C. mannii***
***C. oliveri***	***C. oliveri***	***C. oliveri***	***C. oliveri***	***C. oliveri***
***C. sinensis***	***C. sinensis***	(*C. harringtonii*)	(*C. harringtonii*)	***C. sinensis***
				***C. sp. nov.***

*Species in bold are recognized in each taxonomic classification, the species in bracket indicates that correspond recognized species in other classifications merged into the species.*

Intrageneric phylogenetic relationships of *Cephalotaxus* also remained largely unresolved or disputable in previous studies which did not include all extant lineages or only used a few gene sequences. For example, Zhang et al. (2000) employed AFLP markers to discriminate only four species and four varieties of *Cephalotaxus*. Hao et al. (2008) investigated the interspecific relationships of Taxaceae and Cephalotaxaceae based on four cpDNA regions and the nrDNA ITS region with a complete sampling of 12 species and two varieties of *Cephalotaxus*. In their studies, *Cephalotaxus latifolia* fell on the basal branch sister to the rest of the species resolved as polytomy, with sister relationships of *C. griffithii* and *C. oliveri*, *C. mannii* and *C. hainanensis*, *C. lanceolata* and *C. fortunei*, and a clade *C. harringtonia* (C. *wilsoniana*, *C. koreana, C. harringtonia* cv. *fastigiata*). Ji et al. (2021) reconstructed a phylogeny of *Cephalotaxus* based on 81 plastid protein-coding genes and ten sampled species. Their interspecific relationships were fully resolved with strong support, but differed with that in Hao et al. (2008) despite both being based on plastid DNA loci. The conflicting relationships of *Cephalotaxus* in previous studies need to be verified using more robust molecular markers and more species sampled. While misidentification of samples will also lead to erroneous phylogenetic relationships, a correct species identification is fundamental for phylogeny reconstruction.

The species of *Cephalotaxus* have important economic values and are used as ornamental plants, and also as medicinal plants due to their alkaloids (Abdelkafi and Nay, 2012). A few species are listed as endangered and vulnerable by IUCN^1^. Thus, a correct taxonomy and accurate species delimitation are crucial to the utilization and conservation of extant *Cephalotaxus* species. In this study, we performed genome skimming and assembled the complete plastome of 32 samples, including multiple samples for all *Cephalotaxus* species recognized in past treatments, and covering the genus’ main distribution range. We aimed to: (1) delimit and discriminate the species of *Cephalotaxus* using the entire plastome and a phylogenetic approach using the taxonomic classification of Farjon (2010) as baseline; (2) to compare these results to those obtained using standard DNA barcodes (*mat*K, *rbc*L, *trn*H-*psb*A, and *trn*L-*trn*F); (3) investigate candidates for specific barcodes; (4) based on the plastome tree propose a phylogeny-based species classification; and (5) discuss the importance of correct species identification and classification on conservation assessments for the genus.

**TABLE 2:** Taxa included in this study with locality, voucher, GenBank accession numbers, and NGS performance for the 32 sequenced samples of *Cephalotaxus* (according to Farjon, 2010) and outgroups.

Taxon Farjon, 2010	Taxon (This study)	Locality information	Voucher	Sample ID	GenBank accession numbers	No. of reads after trimming	No. of mapped reads	Sequencing coverage (mean)	Plastome size (bp)	GC content (%)
*C. fortunei* var. *alpina*	*C. alpina*	China, Yunnan, Weixi	GLM-092565	C114	OK138579	15355626	192686	212.9	136,048	35.10%
*C. fortunei* var. *alpina*	*C. alpina*	China, Yunnan, Lanping	LJ-09438	C118	OK138581	17589552	188214	207.5	136,127	35.10%
*C. fortunei* var. *alpina*	*C. alpina*	China, Yunnan, Shilin	LJ-09396	C113	OK138578	25002738	867933	956.9	136,154	35.10%
*C. fortunei* var. *fortunei*	*C. fortunei*	China, Yunnan, Guangnan	GLM-06122	C011	OK138561	15545572	293789	324.4	136,543	35.10%
*C. fortunei* var. *fortunei*	*C. fortunei*	China, Fujian, Jiangle	LIDZ-0723	C014	OK138562	7516066	33080	150.0	136,770	35.10%
*C. fortunei* var. *fortunei*	*C. fortunei*	China, Gansu, Wenxian	Zhdq-194	C018	OK138563	18982656	229359	253.1	136,807	35.10%
*C. hainanensis*	*C. hainanensis*	Vietnam, Quang Binh, Botrach	WP-1005	C124	OK138584	15758028	103779	114.0	136,679	35.00%
*C. hainanensis*	*C. hainanensis*	China, Hainan, Ledong	GLM-07384	C019	OK138564	10744576	44787	49.4	136,705	35.00%
*C. hainanensis*	*C. hainanensis*	China, Hainan, Ledong	GLM-08821	C021	OK138565	9630812	38936	42.6	137,024	35.00%
*C. harringtonii* var. *harringtonii*	*C. harringtonii* var. *harringtonii*	Korea	20-98	3201	OK138560	7005528	91009	100.3	136,049	35.10%
*C. harringtonii* var. *harringtonii*	*C. harringtonii* var. *harringtonii*	Japan	RBGE19687277A	C122	OK138583	8423756	93116	102.6	136,152	35.10%
*C. harringtonii* var. *nana*	*C. harringtonii* var. *nana*	Korea, Seoul, Backnoon Mt.	K-15	C024	OK138566	11120458	65482	72.2	136,196	35.10%
*C. harringtonii* var. *nana*	*C. harringtonii* var. *nana*	Korea, Seoul, Backnoon Mt.	K-11	C030	OK138567	25610494	112136	123.4	136,290	35.10%
*C. harringtonii* var. *wilsoniana*	*C. harringtonii* var. *wilsoniana*	China, Taiwan, Yinlan	MMO-081382	C120	OK138582	16120144	141039	155.3	136,189	35.10%
*C. harringtonii* var. *wilsoniana*	*C. harringtonii* var. *wilsoniana*	China, Taiwan, Xinzhu	GLM-103071	C129	OK138591	15792126	49311	54.3	136,308	35.10%
*C. harringtonii* var. *wilsoniana*	*C. harringtonii* var. *wilsoniana*	China, Taiwan, Nantou	GLM-103119	C132	OK138587	16437696	193905	213.8	136,218	35.10%
*C. lanceolata*	*C. lanceolata*	China, Yunnan, Gongshan	DLJET-660	C031	OK138568	18634280	68790	79.9	136,496	35.00%
*C. lanceolata*	*C. lanceolata*	China, Yunnan, Gongshan	GLM-092527	C116	OK138580	17375238	65088	75.6	136,497	35.00%
*C. latifolia*	*C. sinensis*	China, Chongqing, Nanchuan	86	C105	OK138576	16651598	79425	87.4	136,798	35.10%
*C. latifolia*	*C. sinensis*	China, Chongqing, Nanchuan	131	C107	OK138577	16182814	97845	107.7	136,385	35.10%
*C. mannii*	*C. mannii*	China, Yunnan, Puer	GLM-07382	C038	OK138569	17845098	121796	133.6	136,792	35.10%
*C. mannii*	*C. mannii*	China, Yunnan, Jinghong	GLM-07383	C039	OK138570	17220756	171004	187.7	136,792	35.10%
*C. mannii*	*C. mannii*	China, Yunnan, Hekou, Nanxi	GLM-164317	C133	OK138588	30387206	95923	105.9	136,870	35.10%
*C. mannii*	*C. mannii*	China, Xizang, Motuo	2427	C126	OK138585	16738946	95533	104.8	136,788	35.10%
*C. mannii*	*C. mannii*	China, Xizang, Motuo	ZSD001	C128	OK138586	18521626	133823	146.8	136,811	35.10%
*C. oliveri*	*C. oliveri*	China, Sichuan, Emei Mt.	21	C041	OK138571	15658890	41015	45.7	134,626	35.20%
*C. oliveri*	*C. oliveri.*	China, Chongqing, Nanchuan	84	C043	OK138572	15724158	31618	35.2	134,601	35.20%
*C. oliveri*	*C. oliveri.*	China, Yunnan, Pingbian	GLM-07587	C044	OK138573	19709362	52593	58.5	136,610	35.20%
*C. sinensis*	*C. sinensis*	China, Sichuan, Meigu	GLM-07750	C052	OK138574	17683482	117227	128.7	136,663	35.10%
*C. sinensis*	*C. sinensis*	China, Hunan, Sangzhi	ZhangSD-25	C096	OK138575	25525226	311275	342.4	136,535	35.10%
*C. sinensis*	*C. sp. nov.*	China, Guizhou, Anlong	GLM-06029	C032	OK138589	19382784	36760	40.3	136,929	35.10%
*C. sinensis*	*C. sp. nov.*	China, Yunnan, Xichou	GLM-07336	C037	OK138590	19043154	271634	318.1	137,285	35.10%
*Torreya taxifolia*	*Torreya taxifolia*	United States, GA, Atlanta Botanical Garden	20121291	To33	OK138559	39120638	452291	498.8	137,117	35.40%
*Torreya jackii*	*Torreya jackii*	China. Jiangxi, Fuzhou	PVHJX03233	To43	OK138558	25767386	451825	495	137,117	35.50%
*Amentotaxus formosana*	*Amentotaxus formosana*	China, Taiwan, Pingtung	MMO-081377	Am10	OK138557	24414070	450086	500	136,361	35.80%

## Materials and Methods

### Taxon Sampling

To cover the maximum diversity within species, 2-5 individuals from different populations per species and varieties were sampled in the field for this study. A total of 32 individuals of 8 species and 3 varieties in *Cephalotaxus* following Farjon’s latest classification (Farjon, 2010) were sampled, which also covered all the taxa raised in other recent taxonomic revisions of this genus (e.g., Fu et al., 1999; Lang et al., 2013; Zhang et al., 2019). These samples covered most of the geographic distribution range of *Cephalotaxus*. We selected *Torreya jackii* Chun, *T. taxifolia* Arn and *Amentotaxus formosana* H.L. Li as outgroups (Hao et al., 2008; Ran et al., 2018; Ji et al., 2021). More detailed samples information can be found in Table 2. Healthy leaves were collected and dried immediately in silica-gel for DNA extraction. A few samples were collected from herbarium specimens. Voucher specimens of most sampled taxa were deposited at the Herbarium of Kunming Institute of Botany (KUN), Chinese Academy of Sciences.

### Illumina Sequencing, Assembly, and Annotation

Total genomic DNA was extracted from ∼20 mg of leaf samples by a modified CTAB method (Doyle and Doyle, 1987). Approximately 5 μg of purified genomic DNA was used to construct shotgun libraries with a TruSeq DNA Sample Prep Kit following the manufacturer’s instructions (NEBNext^®^ Ultra IITMDNA Library Prep Kit for Illumina^®^). Paired-end sequencing from both ends of 150 bp fragments was performed on an Illumina HiSeq X Ten platform (Illumina, San Diego, CA, United States) at BGI (Wuhan, China) to generate approximately 3 Gb data for each individual sample. Raw reads were filtered to remove adaptors and low quality reads using the NGS QC Toolkit (Patel and Jain, 2012) with default parameters.

The plastomes were *de novo* assembled from the genome skimming data using the GetOrganelle toolkit (Jin et al., 2020). The complete plastome of *C*. *hainanensis* (NC_042392) was used as the reference. Plastid genes were annotated using PGA (Qu et al., 2019) and coupled with manual adjustment in Geneious v8.0.2 (Kearse et al., 2012). The tRNAs were checked with tRNAscan-SE v2.0.3 (Lowe and Chan, 2016). Final plastid genome map was drawn using OGDRAW (Lohse et al., 2013).

### Data Analysis

We employed the complete plastome sequences for species delimitation using tree-based and genetic distance methods. To compare the discriminatory power of standard DNA barcode and super-DNA barcode (the whole plastome), the standard DNA barcodes of *mat*K, *rbc*L, *trn*H-*psb*A and *trn*L-*trn*F sequences were extracted from the complete plastomes. Besides, DnaSP v6.12 (Rozas et al., 2017) was used to perform a sliding window analysis with a step size of 200 bp and a window length of 800 bp on the alignment of all *Cephalotaxus* species plastomes, to detect the rapidly evolving candidate molecular markers for species delimitation and discrimination. Nucleotide diversity (Pi) was computed for constructing a DNAsp graph.

In tree-based analyses, since some inversions were found in the plastomes of the outgroups (*Torreya jackii*, *T. taxifoli*a and *Amentotaxus formosana*) compared with *Cephalotaxus*, we adjusted the plastid genome structure of the outgroups using Draft in the MULAN web server^2^ (Ovcharenko et al., 2005) with *Cephalotaxus fortunei* (C011) as the reference sequence to make the gene order and direction collinear across the genome for dataset alignment. Sixteen datasets were constructed in this study: (a) the complete plastomes, (b) six based on the standard DNA barcodes and some of their combinations: *mat*K, *rbc*L, *trn*H-psbA, *trn*L-t*rn*F, *mat*K + *rbc*L, and *mat*K + *rbc*L + *trn*H-*psb*A + *trn*L-*trn*F, (c) eight from selected high variation loci: *trn*I-*rrn*16, *ycf*1, *chl*N-*ycf*1, *clp*P-*acc*D, *rps*16, *acc*D, *ycf*2, *ndh*F-*trn*R, and (d) added the 10 sampled species of Ji et al. (2021) to our dataset and used only the 81 plastid protein-coding genes. The complete plastomes and the DNA loci in each datasets were aligned separately using the program MAFFT v7.221 (Katoh and Standley, 2013) with manual adjustments where necessary and concatenated into data matrices. Phylogenetic analyses for each dataset using maximum likelihood (ML) were performed in RAxML v8.2 (Stamatakis, 2014) under the GTRGAMMA model. The best-scoring ML tree for each dataset was produced with 1,000 bootstrap replicates to provide support values for each node. Taxon with multiple individuals resolved as monophyletic with bootstrap support (BS) over 50% were treated as successfully discriminated against Farjon’s (2010) classification.

To assess the barcoding gap, inter- and intraspecific distances were calculated using the Kimura 2-parameter (K2P) distance in MEGA v10.1.8 (Kumar et al., 2018) for each of the 16 data sets. A discrete distribution difference between the average intraspecific and the average interspecific genetic distance is indicative of the existence of a barcoding gap (Hebert et al., 2004).

## Results

### Characteristics of *Cephalotaxus* Plastomes

**FIGURE 1:**
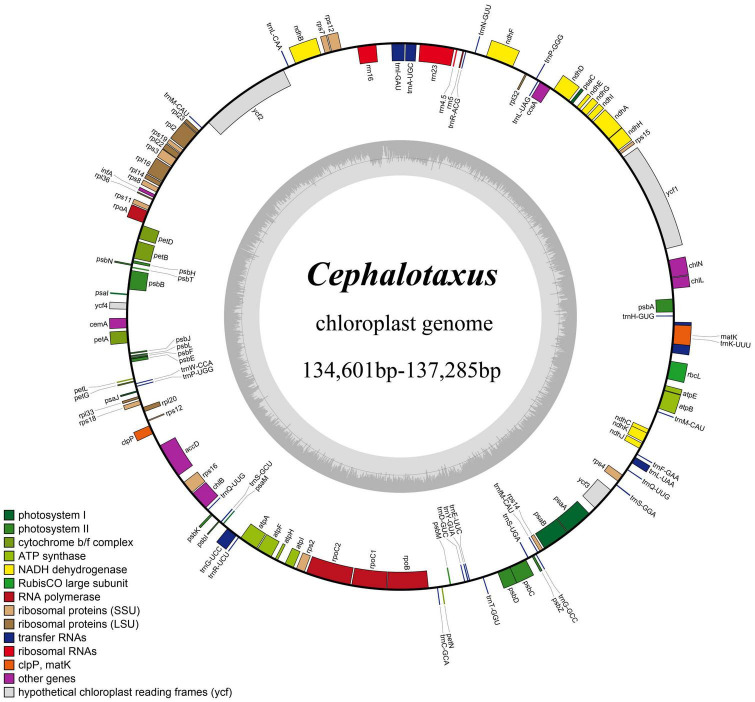
Map of the *Cephalotaxus* plastomes. Genes shown outside the circle are transcribed clockwise, and genes inside are transcribed counterclockwise. The dark gray area in the inner circle indicates the GC content of the plastomes.

High-quality complete plastid genomes of all 32 sampled individuals of *Cephalotaxus* were obtained and assembled into circular molecules (Figure 1 and Table 2). For the 32 individuals, 31,618 (*C*. *oliveri* C043) to 867,933 (*C. fortunei* var. *alpina* C113) reads were mapped to the newly assembled plastomes with an average sequencing depth ranging from 35.2× to 956.9×. The length of the 32 *de novo* assembled *Cephalotaxus* plastomes ranged from 134,601 to 137,285 bp with very similar GC contents (35.1–35.2%) (Table 2). The plastomes of all *Cephalotaxus* samples included 113 unique genes in identical order, comprising 4 rRNA genes, 27 tRNA genes and 82 protein-coding genes (Table 3). Identical to the published *Cephalotaxus* plastomes, an inverted repeat region was lost, resulting in difficulties in defining the boundary between the large and small single-copy regions (Figure 1).

**TABLE 3:** Gene content of the *Cephalotaxus* plastomes.

Gene category	Gene group	Gene name
Self-replication	Ribosomal RNA gene	*rrn*4.5, *rrn*5, *rrn*16, *rrn*23
	Transfer RNA gene	*trn*A-UGC*, *trn*C-GCA, *trn*D-GUC, *trn*E-UUC, *trn*F-GAA, *trn*fM-CAU, *trn*G-GCC, *trn*G-UCC*, *trn*H-GUG, *trn*I-GAU*, *trn*K-UUU*, *trn*L-CAA, *trn*L-UAA, *trn*L-UAG, *trn*M-CAU × 2, *trn*N-GUU, *trn*P-GGG, *trn*P-UGG, *trn*Q-UUG × 2, *trn*R-ACG, *trn*R-UCU, *trn*S-GCU, *trn*S-GGA, *trn*S-UGA, *trn*T-GGU, *trn*W-CCA, *trn*Y-GUA
	Large subunit of ribosome	*rpl*2*, *rpl*14, *rpl*16*, *rpl*20, *rpl*22, *rpl*23, *rpl*33, *rpl*36
	Small subunit of ribosome	*rps*2, *rps*3, *rps*4, *rps*7, *rps*8, *rps*11, *rps*12**, *rps*14, *rps*15, *rps*16*, *rps*18, *rps*19
	RNA polymerase	*rpo*A, *rpo*B, *rpo*C1*, *rpo*C2
	Translational initiation factor	*inf*A
Gene for photosynthesis	Subunits of photosystem I	*psa*A, *psa*B, *psa*C, *psa*I, *psa*J, *psa*M
	Subunits of photosystem II	*psb*A, *psb*B, *psb*C, *psb*D, *psb*E, *psb*F, *psb*H, *psb*I, *psb*J, *psb*K, *psb*L, *psb*M, *psb*N, *psb*T, *psb*Z
	Subunits of NADH dehydrogenase	*ndh*A*, *ndh*B*, *ndh*C, *ndh*D, *ndh*E, *ndh*F, *ndh*G, *ndh*H, *ndh*I, *ndh*J, *ndh*K
	Subunits of cytochrome	*pet*A, *pet*B*, *pet*D*, *pet*G, *pet*L, *pet*N
	Subunit for ATP synthase	*atp*A, *atp*B, *atp*E, *atp*F*, *atp*H, *atp*I
	Large subunit of rubisco	*rbc*L
Other genes	Maturase	*mat*K
	Protease	*chl*B, *chl*L, *chl*N
	Envelope membrane protein	*cem*A
	Subunit of acetyl-CoA carboxylase	*acc*D
	ATP-dependent protease	*clp*P
	C-type cytochrome synthesis gene	*ccs*A
	Open reading frames (ORF, *ycf*)	*ycf*1, *ycf*2, *ycf*3**, *ycf*4*

×2, Two gene copies; *, with one intron; **, with two introns.

### Whole Plastome as Super-Barcode for Discriminating *Cephalotaxus* Species

**FIGURE 2:**
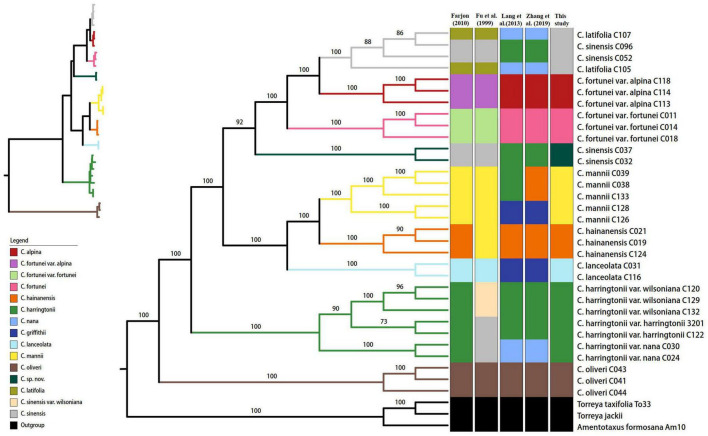
Phylogenetic tree reconstructed via maximum-likelihood (ML) analysis of the complete plastome data. Numbers above branches indicate the bootstrap support values. In the top left is an ML phylogenetic tree with branch length excluding outgroups. The branch colors correspond with the species recognized in this study.

We obtained an alignment matrix of 142,194 bp, which had 3,795 (2.67%) variable sites including 3,467 (2.44%) parsimony informative (PI) sites (Table 4). The ML tree showed that all the taxon nodes (the node grouping individuals from the same species/variety) had maximum support values (BP = 100%), except for *C. harringtonii* var. *harringtonii* (BP = 73%) (Figure 2). Based on the classification of Farjon (2010), five species (*C. hainanensis*, *C. harringtonii*, *C. lanceolata*, *C. mannii*, and *C. oliveri*) out of eight of his recognized species (62.5%) were successfully discriminated with highest support values, while the remaining three, *C. latifolia*, *C. fortunei*, and *C. sinensis*, were non-monophyletic. Within *C. harringtonii*, samples of each variety formed a clade respectively, and *C. harringtonii* var. *nana* was sister to the other two conspecific varieties. Samples of *C. sinensis* (C052 and C096) and *C. latifolia* (C105 and C107) were recovered in a clade (BP = 100%), but neither species was monophyletic. Interestingly, two individuals (C032 and C037) of *C. sinensis* formed a distinct clade as sister to a clade (BP = 100%) that included samples of *C. fortunei*, *C. sinensis* (C052 and C096) and *C. latifolia* (Figure 2). Although samples of the two varieties of *C. fortunei* (*C. fortunei* var. *fortunei*, *C. fortunei* var. *alpina*) each formed a separate clade, they were not monophyletic (Figure 2). The phylogenetic tree including additionally 10 *Cephalotaxus* species used in Ji et al. (2021) based on 81 protein-coding genes recovered the same topology with strong support as the complete plastome tree. In this tree, six out of the ten species grouped in their respective species clades. Two species, *C. griffithii* (= *C. lanceolata*) and *C. nana* (= *C. latifolia*), grouped into our *C. fortunei* var. *alpina* clade, and the other two species, *C. fortunei* and *C. alpina*, fell in the clade of *C. mannii* (Supplementary Figure S1).

**TABLE 4:** Sequence characteristics of the complete plastome, different DNA loci and the combinations for *Cephalotaxus*.

DNA barcode	No. of sites	No. of variable sites	No. of parsimony informative sites
complete plastome	142194	3795 (2.67%)	3467 (2.44%)
*mat*K + *rbc*L + *trn*H-*psb*A + *trn*L-*trn*F	2585	63 (2.44%)	59 (2.28%)
*mat*K + *rbc*L	1479	19 (1.28%)	19 (1.28%)
*mat*K	783	9 (1.15%)	9 (1.15%)
*rbc*L	696	10 (1.44%)	10 (1.44%)
*trn*H-*psb*A	368	16 (4.35%)	16 (4.35%)
*trn*L-*trn*F	738	28 (3.79%)	24 (3.25%)
*trn*I-*rrn*16	2149	472 (21.96%)	403 (18.75%)
*ycf*1	8988	534 (5.94%)	503 (5.60%)
*chl*N-*ycf*1	2066	154 (7.45%)	142 (6.87%)
*clp*P-*acc*D	1144	73 (6.38%)	69 (6.03%)
*rps*16	1079	50 (4.63%)	45 (4.17%)
*acc*D	3330	214 (6.43%)	173 (5.20%)
*ycf*2	7620	223 (2.93%)	194 (2.55%)
*ndh*F-*trn*R	1633	69 (4.23%)	64 (3.92%)

Four species recognized by Lang et al. (2013), Zhang et al. (2019), i.e., *C. griffithii* (C031, C 116, C126, and C128), *C. hainanensis* (C19, C21, C124, C038, C039, and C133), *C. harringtonii* (C120, C122, C129, C132, 3201, C032, C037, C052, and C096), and *C. nana* (C024, C030, C105, and C107) that differed in their taxonomic treatment with Farjon (2010), were not resolved as monophyletic by the complete plastome data; only three species (*C. fortunei*, *C. alpina*, and *C. oliveri*) recognized by Lang et al. (2013), Zhang et al. (2019) were identified successfully. Based on the phylogenetic tree of the entire plastid genome, nine species including a cryptic lineage (*C. sp. nov.* C032/C037) for *Cephalotaxus* were successfully identified and received high support here (Figure 2), which corresponded well with their geographic distribution and morphology. From here on, we used these species names proposed in this study (Table 1).

All the interspecific relationships within *Cephalotaxus* were fully resolved with strong support values (>92%) based on the phylogenetic tree using complete plastome data (Figure 2). *Cephalotaxus oliveri* was on the earliest diverging branch, and *C. harringtonii* formed the subsequent clade which was sister to the rest of the species. The remaining species formed two clades, i.e., *C. lanceolata* (*C. hainanensis* + *C. mannii*), and *C. sp. nov.* (*C. fortunei* (*C. alpina* + *C. sinensis*)).

### Species Discrimination Based on Standard Plastid DNA Barcodes

**FIGURE 3:**
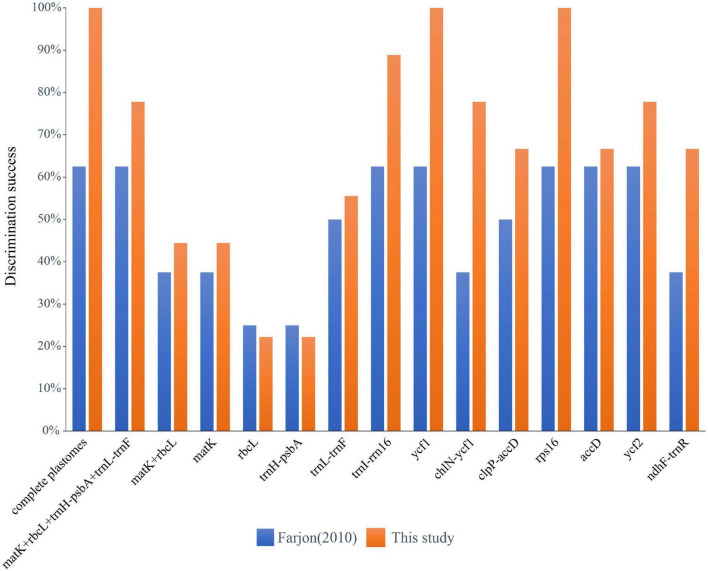
Species discrimination rate of the complete plastome, all single fragments and two DNA barcoding combinations based on the tree-building method.

Among the four single standard DNA barcodes (*rbc*L, *mat*K, *trn*H-*psb*A, *trn*L-*trn*F), *trn*L-*trn*F had the highest number of PI sites of 24 (3.25%, the percentage of informative sites in relation to the length of the sequenced fragment), followed by *trn*H-*psb*A of 16 (4.35%) and *rbc*L of 10 (1.44%), with *mat*K showing the lowest number with 9 (1.15%) (Table 4). The combination of *mat*K + *rbc*L resulted in a matrix of 1479 bp in length, with 19 (1.28%) PI sites, while the combination of the four standard plastid DNA barcodes (*mat*K + *rbc*L + *trn*H-*psb*A + *trn*L-*trn*F) had 2,585 bp and 59 (2.28%) PI sites. Based on the tree-based analysis of the six datasets of the standard DNA barcodes, the single barcodes and combination of *mat*K + *rbc*L distinguished a maximum of four (50%) and five (55.6%) species (*trn*L-*trn*F) for the classification of Farjon (2010) and the one proposed in this study respectively. However, the four-barcode combination (*mat*K + *rbc*L + *trn*H-*psb*A + *trn*L-*trn*F) resolved five (62.5%) and seven (77.8%) species, for the respective classifications (Figure 3 and Supplementary Figure S2).

### Species Discrimination Based on Specific DNA Barcodes

**FIGURE 4:**
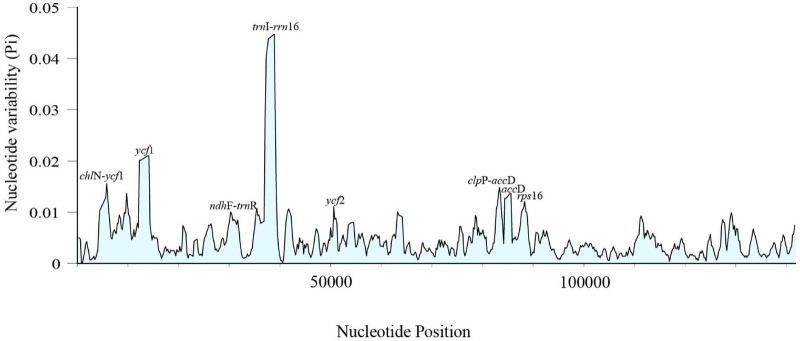
Sliding window analysis of 32 *Cephalotaxus* plastomes (window length: 800 bp, step size: 200 bp). X-axis: position of the midpoint of a window; Y-axis: nucleotide diversity of each window.

Sliding window analyses for all the newly assembled *Cephalotaxus* plastid genomes identified eight highly variable regions (mutational hotspots) (*trn*I-*rrn*16, *ycf*1, *chl*N-*ycf*1, *clp*P-*acc*D, *rps*16, *acc*D, *ycf*2, *ndh*F-*trn*R) (Figure 4). All eight hypervariable specific loci provided higher (maximally 62.5%) or the same (37.5%: *clp*P-*acc*D and *ndh*F-*trn*R showed the same discriminative power as *mat*K, and *mat*K + *rbc*L) species resolution than any single standard DNA barcode or the combination of *mat*K + *rbc*L for the classification of Farjon (2010) (Figure 3). Discrimination success was always higher for the specific loci for the classification proposed in the present study. For this classification, out of these eight specific loci, two, *ycf*1 and *rps*16, yielded the maximum species discriminatory rate, identical to that of the complete plastome, followed by *trn*I-*rrn*16 which identified eight out of nine (89%) species of *Cephalotaxus* (Figure 3 and Supplementary Figure S2).

### Barcoding Gaps

The intraspecific and interspecific K2P distance varied among the 16 datasets. Out of these, the four standard DNA barcodes and their combination, plus *ycf*2, had an interspecific distance of zero, and *chl*N*-ycf*1 the highest intraspecific distance with 2.41%. Although there was some degree of overlapping between the minimum interspecific distance and the maximum intraspecific distance of these loci/combinations (Supplementary Table S1), relatively distinct barcoding gaps were identified for the complete plastome, plastid protein-coding genes and *rps*16, *ndh*F*-trn*R and *ycf*2 (Supplementary Figure S3). Out of these, four datasets, i.e., the complete plastome, plastid protein-coding genes, *ycf*1, and *rps*16, showed maximum species resolution.

## Discussion

### Performance of the Super-, Standard, and Specific Barcodes

With the ever more cost-effective NGS technology, the complete plastid genome assembled from genome skimming data has been used several times as super-barcode with promising potential for plant species delimitation (Kane et al., 2012; Turner et al., 2016; Tonti-Filippini et al., 2017; Fu et al., 2019; Ji et al., 2019; Song et al., 2020). In the present study, compared to the standard barcodes, the complete plastome showed much higher species discrimination rates, and distinguished five out of the eight (62.5%) species of *Cephalotaxus* recognized by Farjon (2010). Only two (25%) to four (50.0%) species were resolved by any single barcode and the combination of *mat*K + *rbc*L, while the combination of all four standard barcodes recovered five species (62.5%), the same as the whole plastome. With view to the nine species proposed here, that all species were highly supported in the phylogeny based on the entire plastomes and the plastid coding genes, the standard DNA barcodes (single barcode and their combinations) only discriminated a maximum of seven (77.8%) of those species (with the combination of *mat*K + *rbc*L + *trn*H-*psb*A + *trn*L-*trn*F) and with relatively low support values (Figure 3 and Supplementary Figure S2).

Our species discrimination results indicated that the complete plastome can provide more variation to discriminate *Cephalotaxus* species compared to the standard barcodes, which has also been demonstrated in other studies (e.g., Ji et al., 2020; Song et al., 2020; Fu et al., 2022). In addition, the complete plastome approach based on genome skimming can circumvent issues of locus choice, and low PCR and sequencing recovery rate, which are sometimes encountered in standard DNA barcoding studies (Ruhsam et al., 2015; Curci et al., 2016). Therefore, the whole plastome as super-barcode illustrated the benefits of moving beyond standard barcodes in *Cephalotaxus* (Tonti-Filippini et al., 2017; Nevill et al., 2020).

Although the super-barcode effectively distinguished species beyond the standard barcodes, its use may sometimes be limited due to the relatively higher costs and complex analyses of genome skimming data compared to the standard PCR and Sanger sequencing barcode approach. A “specific” barcode is a fragment of DNA sequence that has a sufficiently high mutation rate to enable high species identification within a given taxonomic group beyond the standard DNA barcodes, and a more wider use of plant group-specific barcodes for discriminating closely related species was predicted (Li et al., 2015). In this study, we identified eight highly variable loci from the plastid genomes of *Cephalotaxus*. Among them, although *trn*I-*rrn*16 was the most divergent locus, it achieved only a discriminatory rate of 8 out of 9 species (88.9%) (Figure 3), which implied that the selection of barcoding loci based on maximum sequence divergence may not be in line with maximum species discrimination ability. The two loci *ycf*1 and *rps*16 showed the maximum species discrimination ability, the same as the complete plastome (Figure 3). Considering the length of *ycf*1 (8988 bp), it is not suitable as a hypervariable specific barcode. With a PCR-manageable length of 1079 bp, *rps*16 can be proposed here as a specific barcode for discriminating *Cephalotaxus* species. In addition, these identified highly variable loci represent valuable genetic resources for future population genetics and phylogeographic studies.

### Monophyly of Species and Discovery of Cryptic Species

The inclusion of multiple individuals from a single species sampled is highly important to confirm species delimitation successfully and accurately (Liu et al., 2012; Fu et al., 2022). Therefore, in this study, all species/varieties of *Cephalotaxus* had more than one individual included (two to five individuals per taxon). Samples of five species and all varieties in Farjon’s (2010) taxonomic classification were resolved as monophyletic with strong support in the complete plastome phylogeny, while another three species, *C. latifolia*, *C. sinensis* and *C. fortunei*, were not individually resolved as monophyletic. Samples of *C. latifolia* and *C. sinensis* together formed a highly supported clade, but were not resolved in their respective species monophyly. This indicated that they belonged to the same genetic lineage, and suggested a merging of *C. latifolia* into *C. sinensis*. This is supported by their sympatric distribution and overlapping of some morphological traits. Notably, the two samples (C032 and C037) collected from the karst region of southwest Guizhou and southeast Yunnan, China, identified as *C. sinensis* under the classification of Farjon (2010), were recovered in a highly supported isolated clade, suggesting a cryptic species of *Cephalotaxus* (Figure 2). In a study including more individuals collected from this karst region, in adjacent areas in southwest Guizhou, southeast Yunnan and north Vietnam, standard DNA barcodes already indicated the existence of this cryptic species, though it was not distinguished due to a lack of sufficient sequence variations in the nrDNA ITS region (Gao et al., 2015). The new taxon may be adapted to the special karst habitats and has become a distinct species over time, as also found in *Taxus* (Liu et al., 2011; Möller et al., 2013, Möller et al., 2020) and *Amentotaxus* (Gao et al., 2017, 2019) in this region. More detailed morphological study for this group is needed to describe the new species. *Cephalotaxus fortunei* var. *fortunei* and *C. fortunei* var. *alpina* were each resolved as monophyletic respectively, but the species was not monophyletic. This supports the view that the two varieties are best represented at species rank (Lang et al., 2011, 2013; Zhang et al., 2019).

The 81 protein-coding gene phylogenetic tree that included all ten species of *Cephalotaxus* used in Ji et al. (2021) showed that only six of the ten species (*C. oliveri C. harringtonii*, *C. wilsoniana*, *C. hainanensis*, *C. mannii*, and *C. sinensis*) grouped into the respective species clade. However, four species (*C. fortunei, C. alpina, C. griffithii, C. nana*) fell into the clade of other species. For instance, two species, *C. fortunei* (MT555080) and *C. alpina* (MT555079) grouped together with *C. mannii* (MT555084) with very short branch length within *C. mannii* clade, indicating that the samples identified as *C. fortunei* and *C. alpina* in Ji et al. (2021) are misidentified and are likely *C. mannii*. Similarly, *C. griffithii* (MT555081) and *C. nana* (MT555085) were placed in the *C. fortunei* var. *alpina* clade, hinting they were also misidentified and likely represent *C. alpina* (Supplementary Figure S1). According to the geographic distribution of the four species, their identification is sound. For example, *C. griffithii* (MT555081) treated as *C. lanceolata* in Farjon (2010) was collected from Dulongjiang, Gongshan, Yunnan, its type locality. *Cephalotaxus fortunei* was from Nanchuan, Chongqing, and *C. nana* from west Hubei treated as *C. latifolia* in Fu et al. (1999), Farjon (2010). The unusual placement of the four species might be the result from experimental and/or identification errors.

*Cephalotaxus oliveri* has a distinct morphology and no taxonomic controversy in all classifications (Farjon, 1998, 2010; Lang et al., 2011, 2013; Zhang et al., 2019), and the species can be discriminated in all barcoding datasets. Our results did not support the merging of *C. lanceolata* into *C. griffithii*, *C. sinensis* into *C. harringtonii* (Lang et al., 2011, 2013; Zhang et al., 2019), and *C. mannii* into *C. harringtonii* (Lang et al., 2013) or *C. hainanensis* (Zhang et al., 2019), nor supported *C. harringtonii* var. *wilsoniana* as a variety of *C. sinensis* (Fu et al., 1999). *Cephalotaxus lanceolata* formed a distinct clade separate from *C. griffithii* (C126 and C128), and morphologically differed from *C. griffithii* by lanceolate leaves and rounded base. Within *C. harringtonii*, each of the three varieties and the species itself were resolved as monophyletic. The variety *C. harringtonii* var. *wilsoniana* is endemic to Taiwan, China, the other two taxa mainly occur in Japan and Korea. We observed a low sequence variation within the species and samples were missing of *C. harringtonii* var. *nana* from Japan. Therefore, we refrained from a decision of whether to raise the varieties to species level until more samples of this species particularly from Japan are studied.

### Congruence With Previous Phylogenetic Relationships

Phylogenetic relationships of *Cephalotaxus* in previous studies were not fully resolved likely due to a low number of DNA loci used, incomplete sampling, or both (e.g., Cheng et al., 2000; Hao et al., 2008). In this study, the interspecific phylogenetic relationships of *Cephalotaxus* were fully resolved (Figure 2), and the topology is basically consistent with the phylogeny based on 81 plastid protein-coding genes (Ji et al., 2021), but with some differences in interspecific relationships possibly due to experimental error or misidentification (see above). In the present study, *C. oliveri* was on the first diverging branch. *C. harringtonii* formed the subsequent grade and sister to the rest of the species, which was also recovered in Ji et al. (2021), but such relationship was not recovered by the combination of four plastid loci (*mat*K, *rbc*L, *trn*L-F, and *psb*A-*trn*H) and nrITS region (Hao et al., 2008). The remaining species formed two clades in this study, i.e., *C. lanceolata* (*C. hainanensis* + *C. mannii*), and *C. sp. nov.* (*C. fortunei* (*C. alpina* + *C. sinensis*)). The sister relationship of *C. hainanensis* and *C. mannii*, and monophyly of *C. harringtonii* was supported in Hao et al. (2008), but other interspecific relationships were not recovered. While in Ji et al. (2021), *C. alpina* and *C. latifolia* (representing *C. nana* in Ji et al., 2021) were resolved as sister and then as sister to *C. sinensis*. Another difference is represented by *C. fortunei*; this species grouped in the clade of *C. hainanensis* (*C. mannii* (*C. lanceolata* + *C. fortunei*)) being sister to *C. lanceolata* (with low branch support 75%) with very short branch length (Ji et al., 2021). Such inconsistent relationships were likely either due to species misidentification or experimental errors (see above). Therefore, the use of the entire plastome and multiple samples per species from different populations resulted in a fully resolved and accurate phylogeny of *Cephalotaxus*.

### Implications for Conservation

Accurate species delimitation and identification are key to proper species management and conservation (Trias-Blasi and Vorontsova, 2015). Because of the morphological similarities between *Cephalotaxus* taxa, it is often difficult to unambiguously identify species. In this study, the whole plastome as super-barcode in combination with a revised classification enabled an accurate delimitation of taxa, including disputed ones in *Cephalotaxus*, and suggested the presence of a cryptic species. In this genus, *C. lanceolata* and *C. hainanensis* are listed as endangered, *C*. *mannii* and *C*. *oliveri* are vulnerable in the global IUCN Red List (see text footnote 1). Of these, only *C*. *oliveri* is recognized in all classifications (Table 1). In recent taxonomic revisions of the genus (Lang et al., 2013; Zhang et al., 2019), *C. lanceolata* and *C*. *mannii* were merged into *C. griffithii* and *C. hainanensis* respectively, and their conservation rank changed consequently. However, these taxonomic treatments were not supported by our results, and we propose to retain these species. This has conservation consequences. For example, *C. lanceolata* is a species with extremely small populations which are confined to a small area in northwest Yunnan and the adjacent region of north Myanmar, and it is highly endangered. If *C. lanceolata* would be treated as a synonym of *C. griffithii* (Lang et al., 2011, 2013; Zhang et al., 2019), the populations in Yunnan and Myanmar would no longer receive attention for conservation since *C. griffithii* is not listed as endangered species. Our study also discovered a new cryptic species (*C. sp. nov.*). Based on IUCN criteria v3.1 (IUCN, 2001), this cryptic species would be assessed as threatened species due to small population size fewer than 2,500 mature individuals, ongoing habitat degradation from surrounding agriculture and forest clearances in this region. While plants of this taxon had always been identified as *C. sinensis*, a species categorized as Least Concern (LC), and never considered endangered.

## Conclusion

In this study, we have evaluated the species discrimination efficiency of a super-barcode (the complete plastome), standard DNA and specific DNA barcode using 32 plastomes from all recognized species/varieties in different taxonomic classifications of *Cephalotaxus* with multiple samples per taxon. Our phylogenetic results based on the complete plastome sequences indicated that nine species, including a cryptic taxon, could be discriminated phylogenetically and the interspecific relationships of *Cephalotaxus* were fully resolved with strong support. The standard barcodes, alone or in combinations, were unable to discriminate all nine species. Of the eight selected hypervariable specific barcode loci, we found that only *ycf*1 and *rps*16 alone were able to distinguish all nine species, and, because of its PCR-convenient length, propose *rps*16 as a specific barcode. In addition, our result indicates that the inclusion of multiple samples per species from different population is necessary for both plant DNA barcoding and phylogeny studies. The new insights into the species delineation of *Cephalotaxus* in this study will facilitate their identification and would allow targeted conservation management of the endangered *Cephalotaxus* species in the future.

## Data Availability Statement

The original contributions presented in the study are publicly available. This data can be found here: National Center for Biotechnology Information (NCBI) BioProject database under accession numbers BankIt2499120 AM10 OK138557, BankIt2499138 To43 OK138558, BankIt2499138 To33 OK138559, BankIt2499502 3201 OK138560, BankIt2499502 C011 OK138561, BankIt2499502 C014 OK138562, BankIt2499502 C018 OK138563, BankIt2499502 C019 OK138564, BankIt2499502 C021 OK138565, BankIt2499502 C024 OK138566, BankIt2499502 C030 OK138567, BankIt2499502 C031 OK138568, BankIt2499502 C038 OK138569, BankIt2499502 C039 OK138570, BankIt2499502 C041 OK138571, BankIt2499502 C043 OK138572, BankIt2499502 C044 OK138573, BankIt2499502 C052 OK138574, BankIt2499502 C096 OK138575, BankIt2499502 C105 OK138576, BankIt2499502 C107 OK138577, BankIt2499502 C113 OK138578, BankIt2499502 C114 OK138579, BankIt2499502 C116 OK138580, BankIt2499502 C118 OK138581, BankIt2499502 C120 OK138582, BankIt2499502 C122 OK138583, BankIt2499502 C124 OK138584, BankIt2499502 C126 OK138585, BankIt2499502 C128 OK138586, BankIt2499502 C132 OK138587, BankIt2499502 C133 OK138588, BankIt2499536 C032 OK138589, BankIt2499536 C037 OK138590, and BankIt2500849 C129 OK138591.

## Author Contributions

L-MG and D-ZL designed the experiments. L-MG and MM collected the plant samples. JW, C-NF, and Z-QM performed the experiments and analyzed the data. JW, L-MG, and MM wrote the manuscript. All authors read and approved the final manuscript.

## Conflict of Interest

The authors declare that the research was conducted in the absence of any commercial or financial relationships that could be construed as a potential conflict of interest.

## Publisher’s Note

All claims expressed in this article are solely those of the authors and do not necessarily represent those of their affiliated organizations, or those of the publisher, the editors and the reviewers. Any product that may be evaluated in this article, or claim that may be made by its manufacturer, is not guaranteed or endorsed by the publisher.

## Footnotes

1
https://www.iucnredlist.org/search?query=Cephalotaxus&searchType=species

2
http://mulan.dcode.org

## Supplementary Material

Supplementary Figure S1 Maximum Likelihood phylogenetic tree of 42 *Cephalotaxus* samples including the 10 species used in Ji et al. (2021) based on 81 plastid protein-coding genes with proportional branch lengths. Numbers to the right of nodes indicate bootstrap support values.

Supplementary Figure S2 Maximum Likelihood phylogenetic trees of 32 *Cephalotaxus* samples based on the eight highly variable plastid loci, the four single standard DNA barcode and two combinations of standard DNA barcodes (a. *trn*I-*rrn*16, b. *ycf*1, c. *chl*N-*ycf*1, d. *clp*P-*acc*D, e. *rps*16, f. *acc*D, g. *ycf*2, h. *ndh*F-*trn*R, i. *mat*K + *rbc*L + *trn*H-*psb*A + *trn*L-*trn*F, j. *mat*K, k. *mat*K + *rbc*L, l. *rbc*L, m. *trn*H-*psb*A, n. *trn*L-*trn*F). Numbers to the right of nodes indicate bootstrap support values.

Supplementary Figure S3 Relative distribution of intraspecific and interspecific distances for the datasets of the complete plastome, 81 plastid protein-coding genes, combination of the four standard barcodes, and eight highly variable regions (*trn*I-*rrn*16, *ycf*1, *chl*N-*ycf*1, *clp*P-*acc*D, *rps*16, *acc*D, *ycf*2, *ndh*F-*trn*R). x-axes relate to K2P distances arranged in intervals, and the y-axes correspond to the percentage of occurrences.

Supplementary Table S1 Examination of the 15 DNA loci used for discriminating the recognized species of *Cephalotaxus* in this study.
