# Supplementary figures and images for: Testing the Complete Plastome for Species Discrimination, Cryptic Species Discovery and Phylogenetic Resolution in Cephalotaxus (Cephalotaxaceae)
Source: Front Plant Sci. 2022 May 4;13:768810. doi: 10.3389/fpls.2022.768810 (PMC9116380; doi:10.3389/fpls.2022.768810)

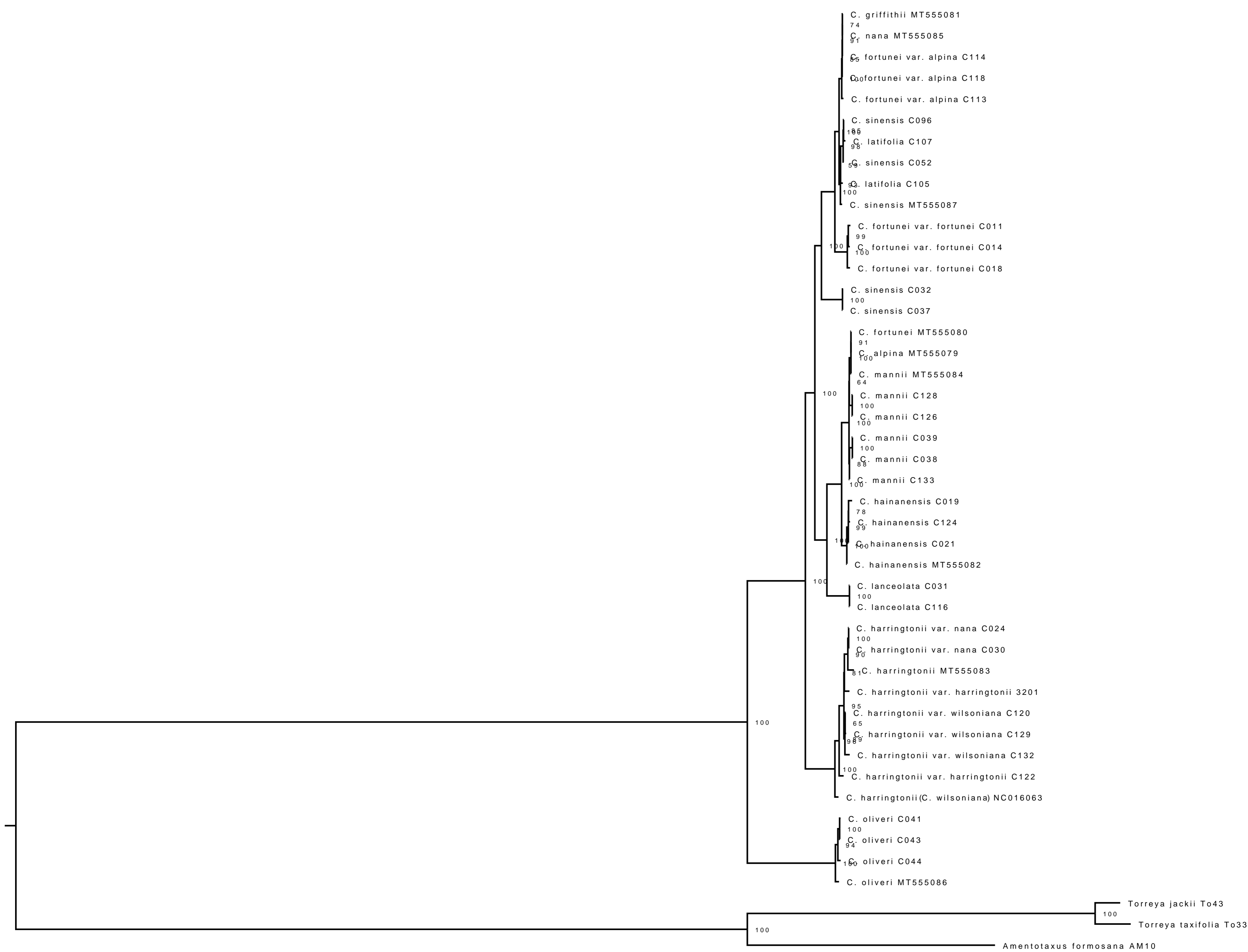

0.007

Supplement: Supplementary Figure S1 — Maximum Likelihood phylogenetic tree of 42 Cephalotaxus samples including the 10 species used in Ji et al. (2021) based on 81 plastid protein-coding genes with proportional branch lengths. Numbers to the right of nodes indicate bootstrap support values. [file Data_Sheet_1.PDF]

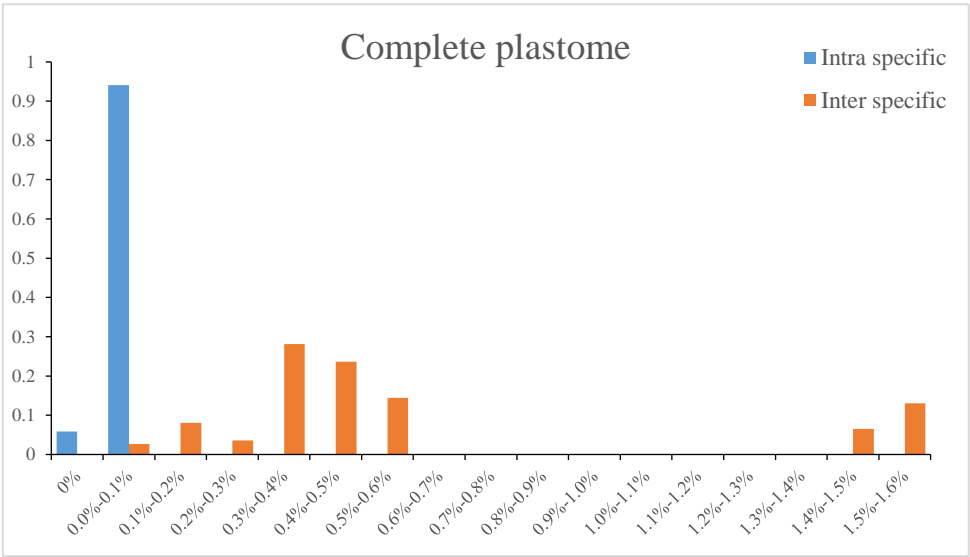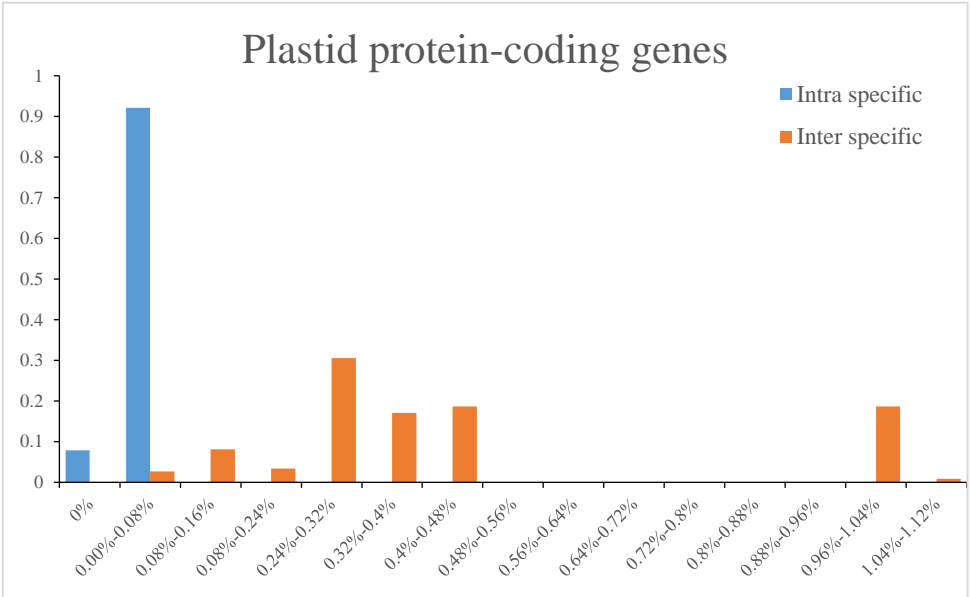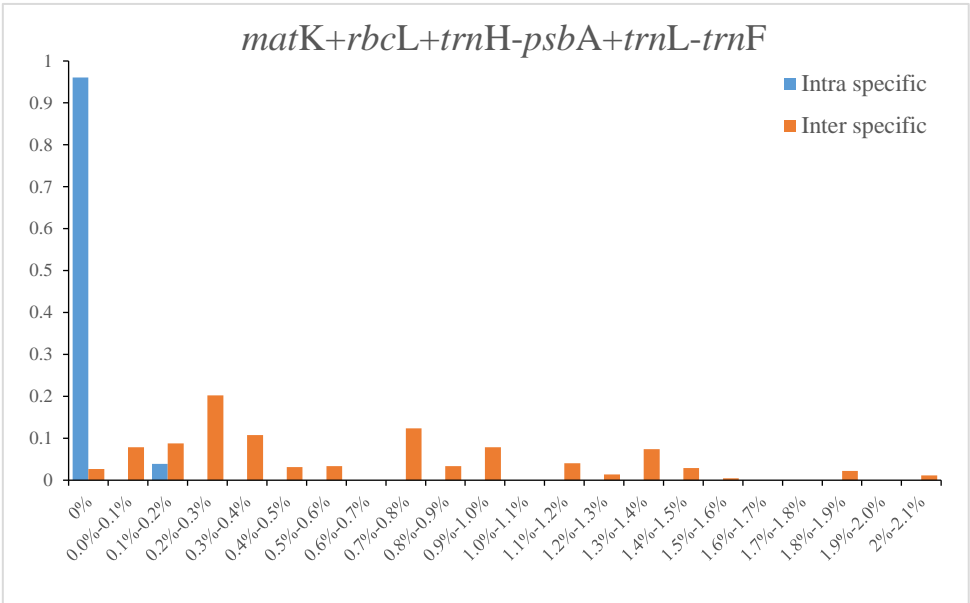

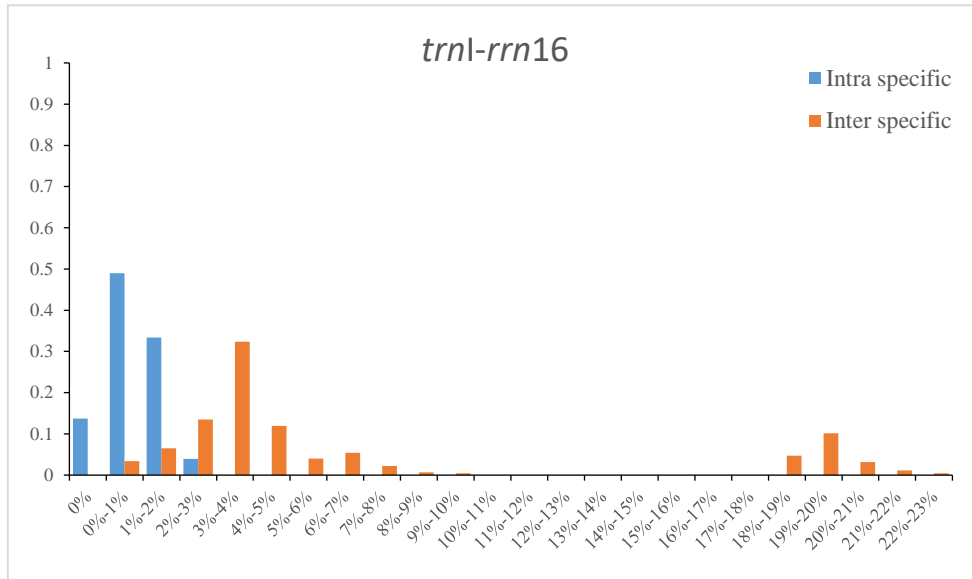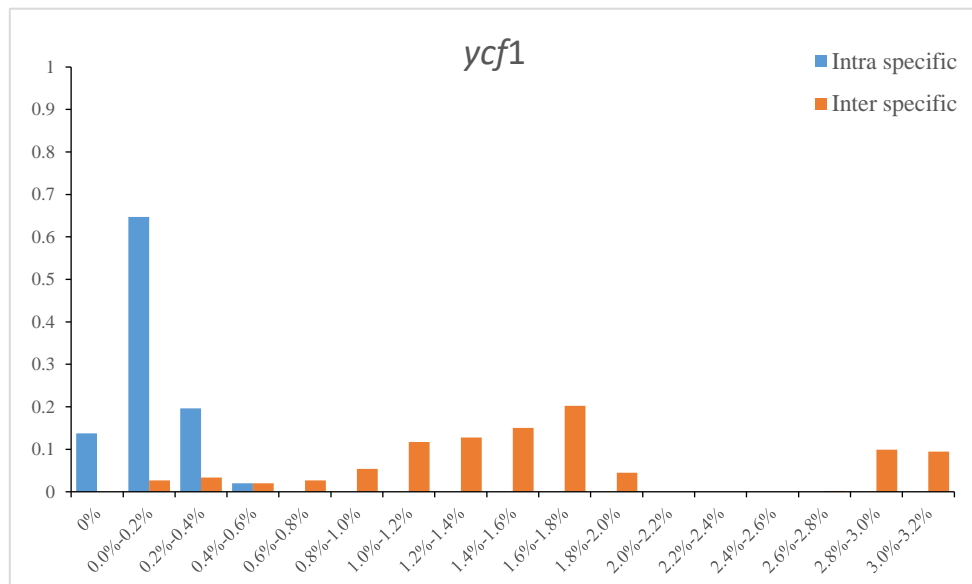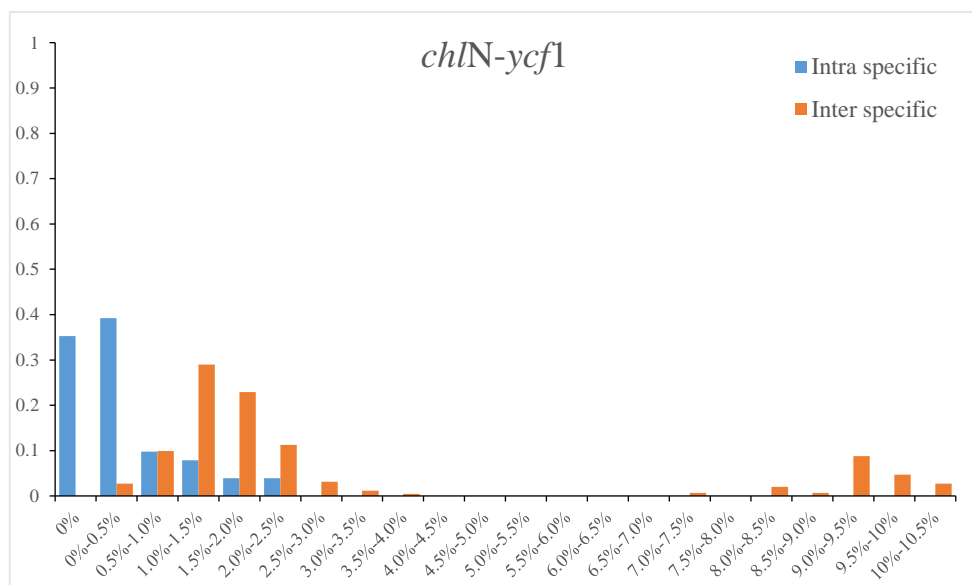

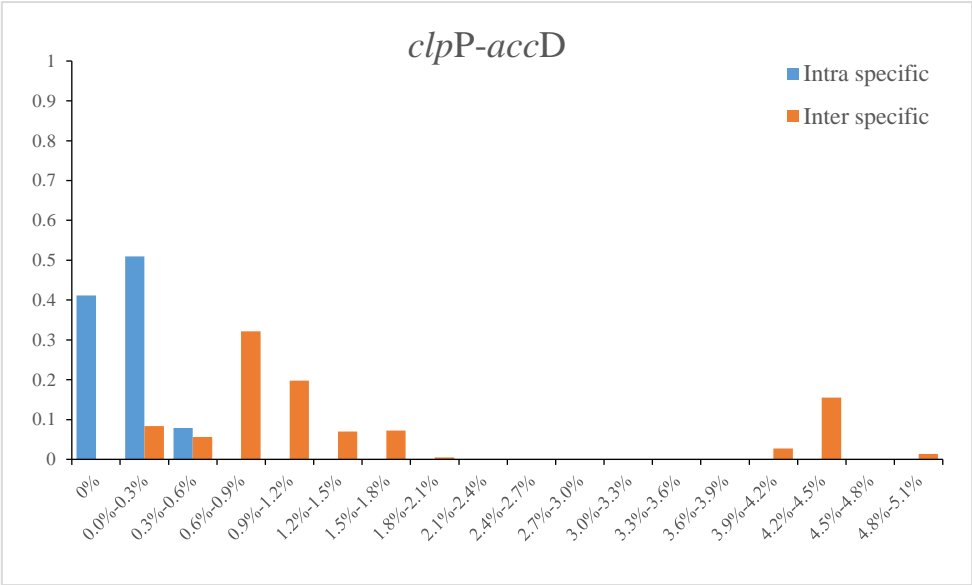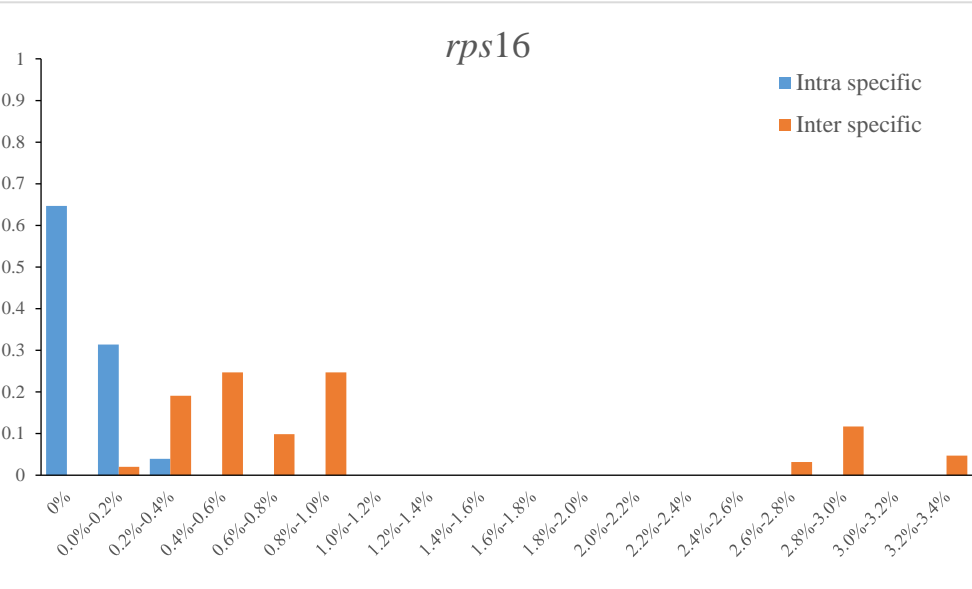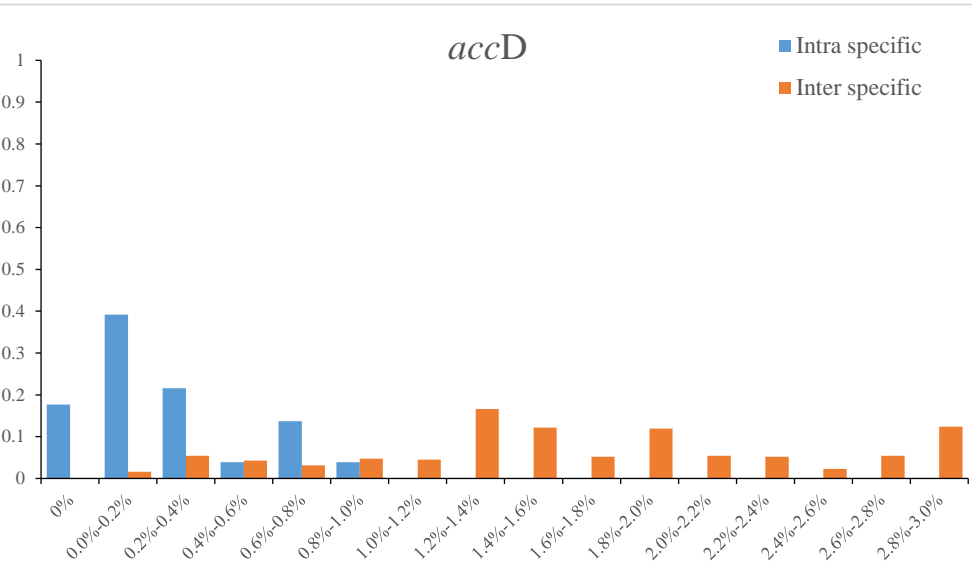

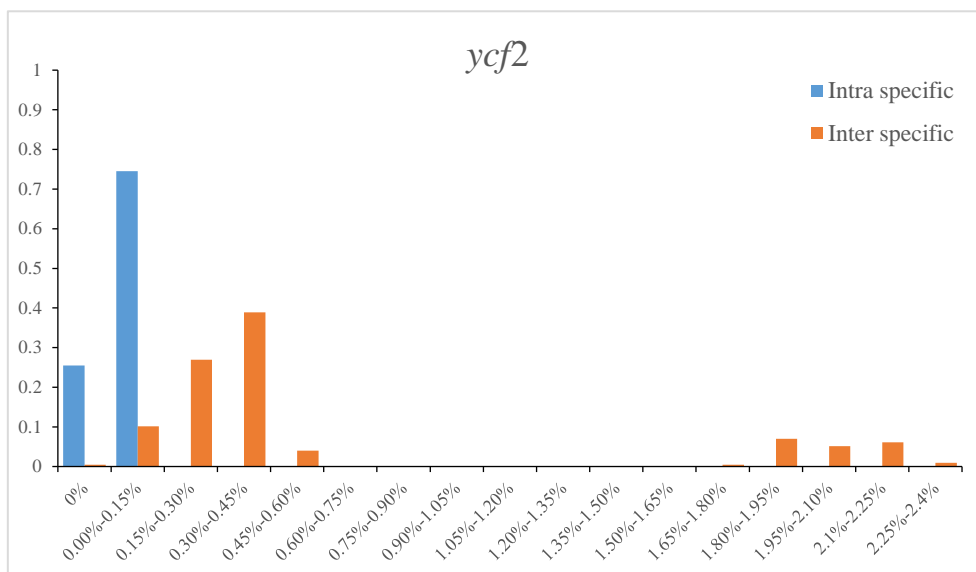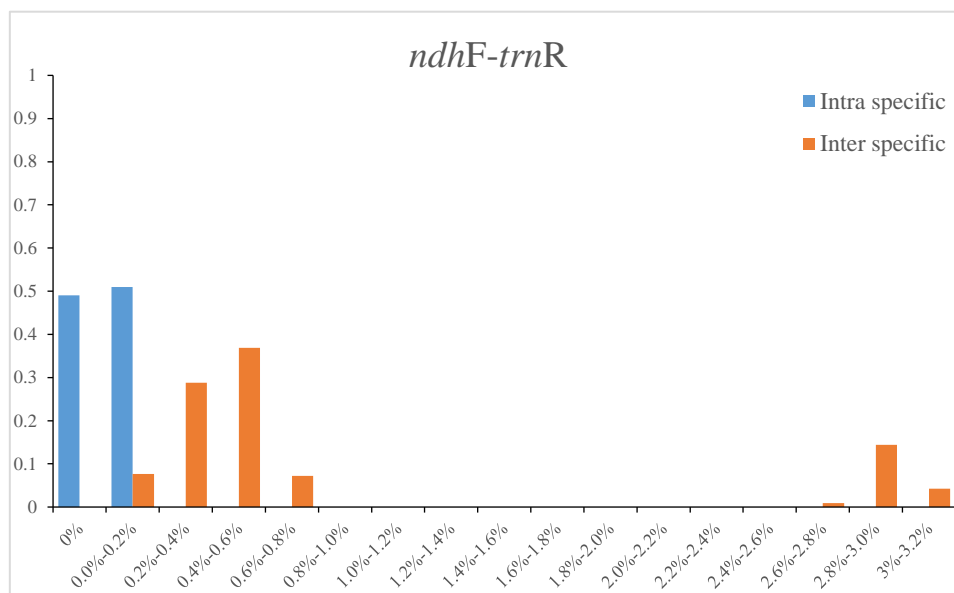

Supplement: Supplementary Figure S3 — Relative distribution of intraspecific and interspecific distances for the datasets of the complete plastome, 81 plastid protein-coding genes, combination of the four standard barcodes, and eight highly variable regions (trnI-rrn16, ycf1, chlN-ycf1, clpP-accD, rps16, accD, ycf2, ndhF-trnR). x-axes relate to K2P distances arranged in intervals, and the y-axes correspond to the percentage of occurrences. [file Data_Sheet_3.PDF]
