# Supplementary material for: Testing the Complete Plastome for Species Discrimination, Cryptic Species Discovery and Phylogenetic Resolution in Cephalotaxus (Cephalotaxaceae)
Source: Front Plant Sci. 2022 May 4;13:768810. doi: 10.3389/fpls.2022.768810 (PMC9116380; doi:10.3389/fpls.2022.768810)

a: *trn*I-*rrn*16

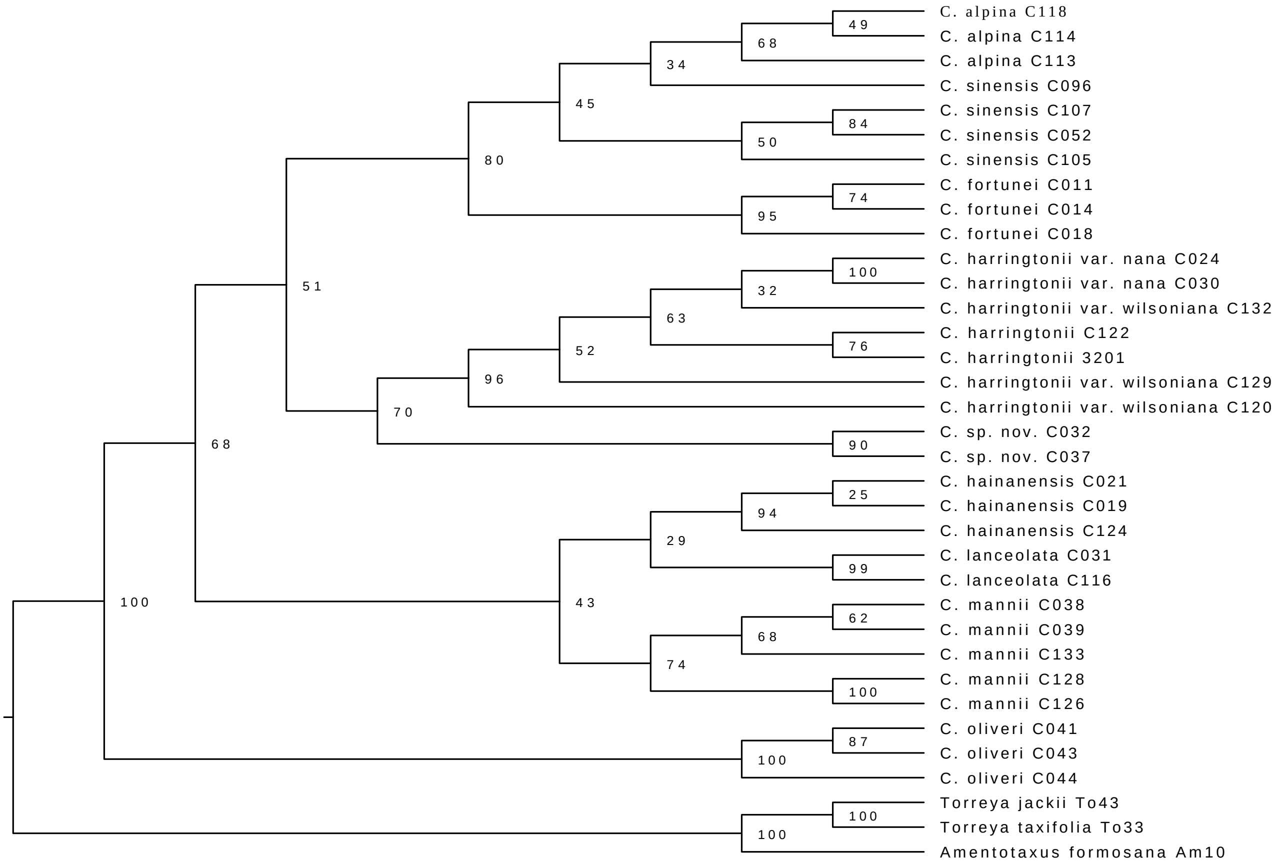

b: *ycf*1

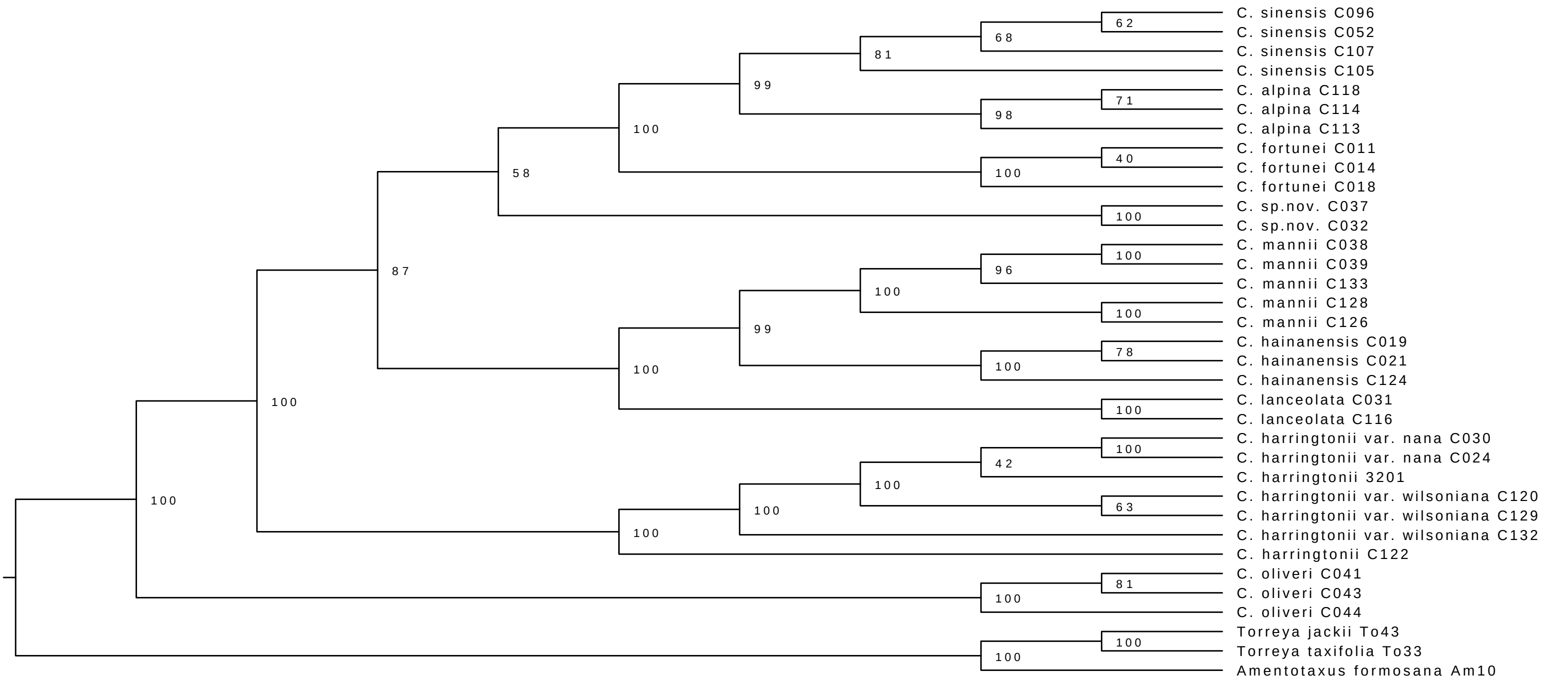

c: *chl*N-*ycf*1

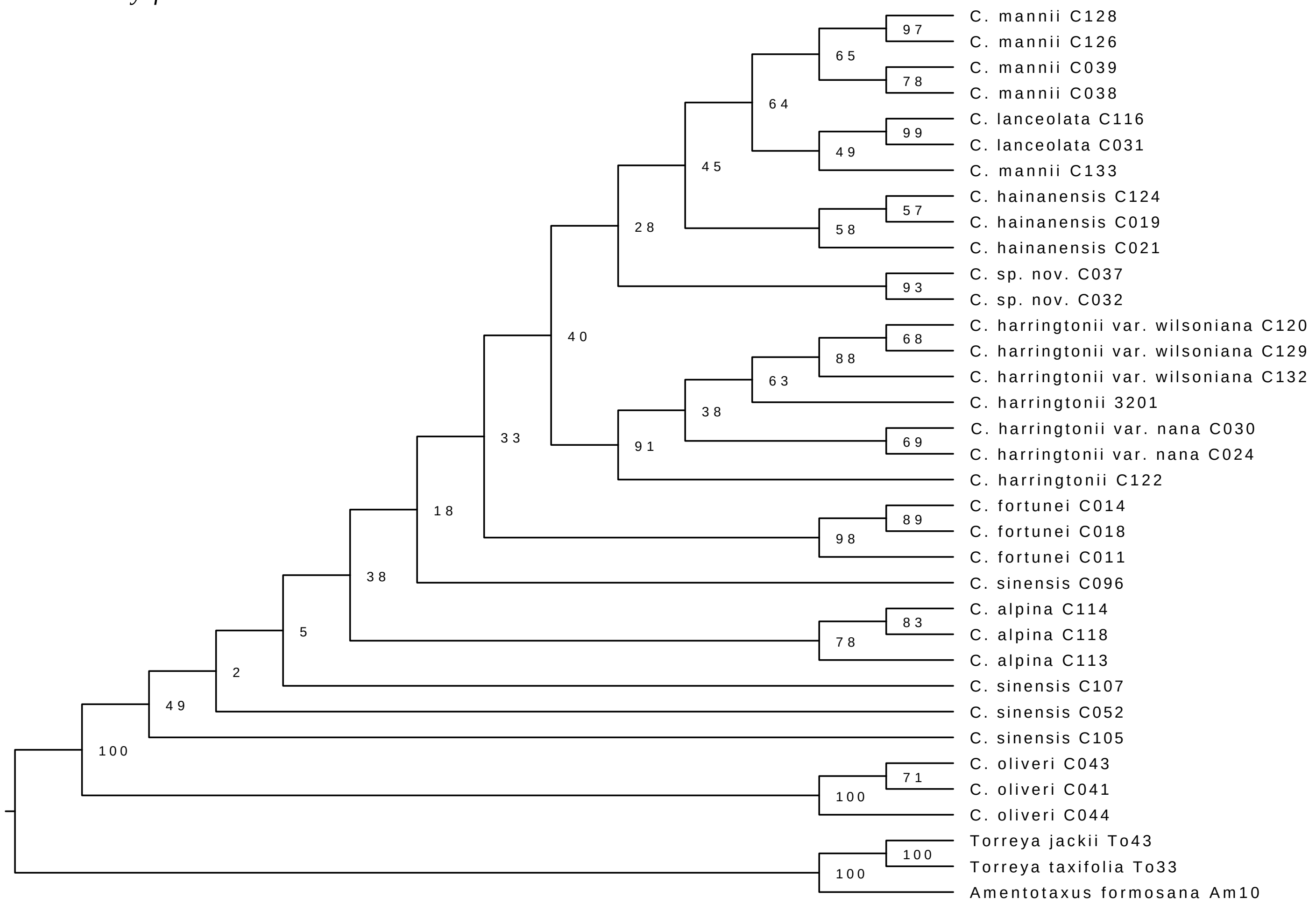

d: *clp*P-*acc*D

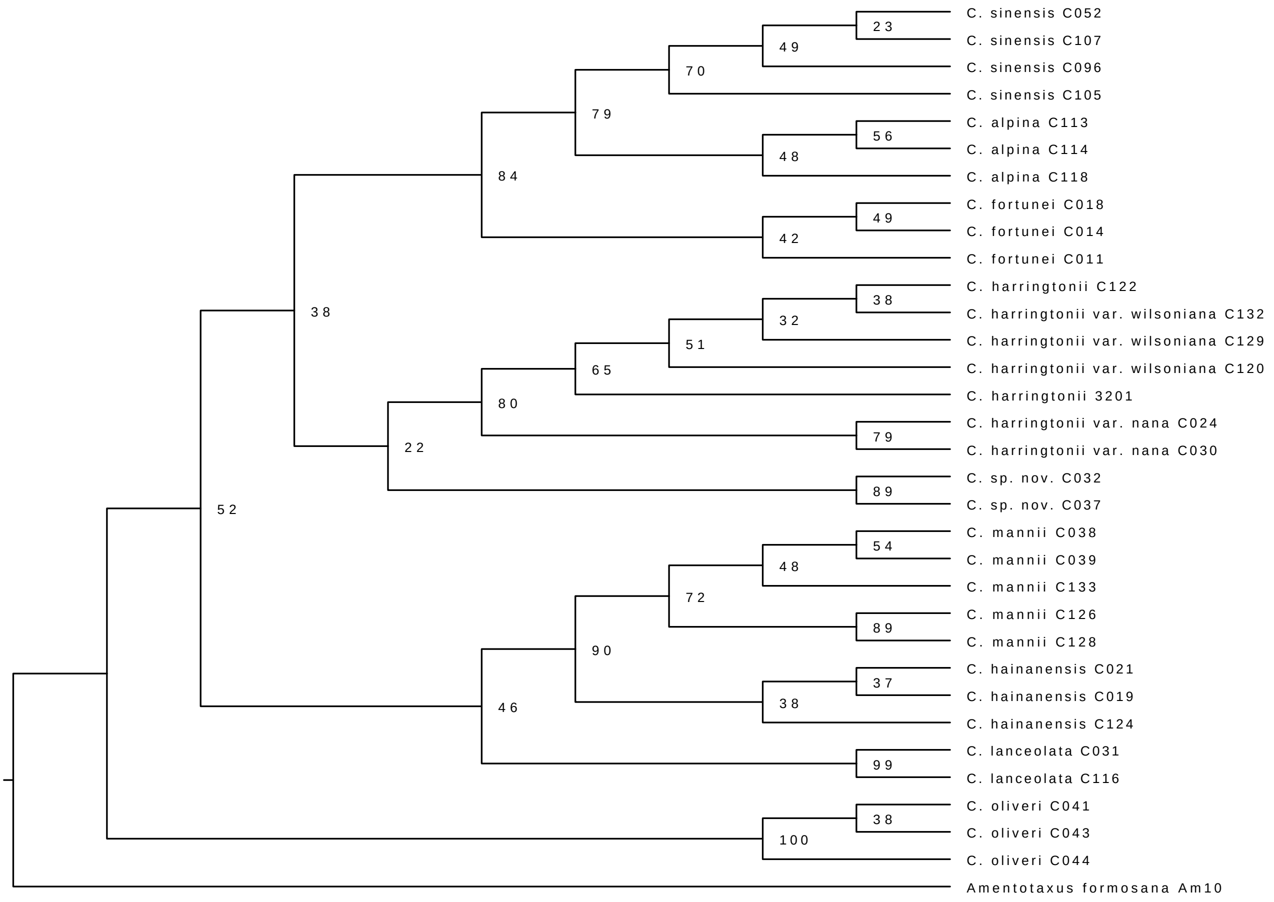

e: *rps*16

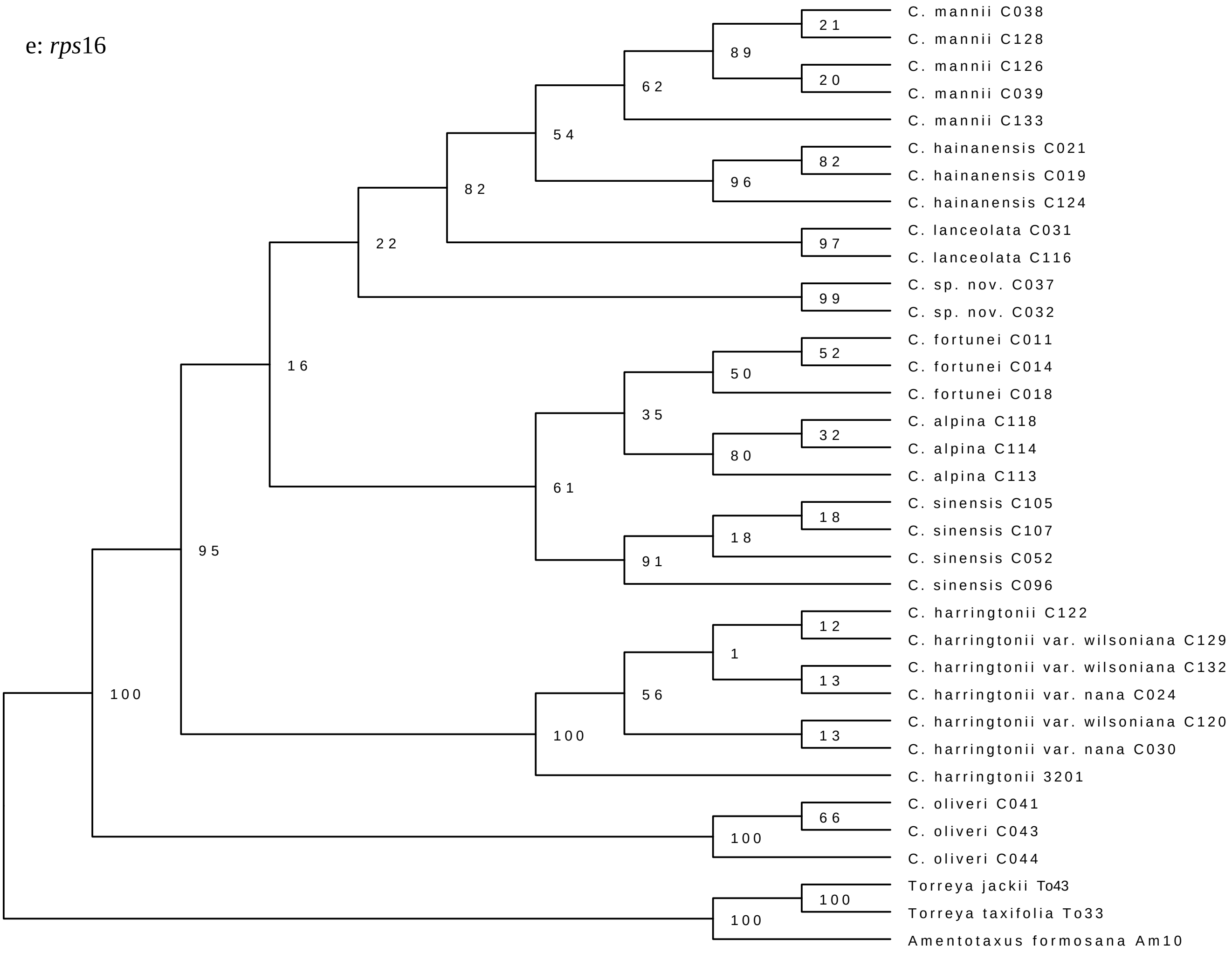

f: *acc*D

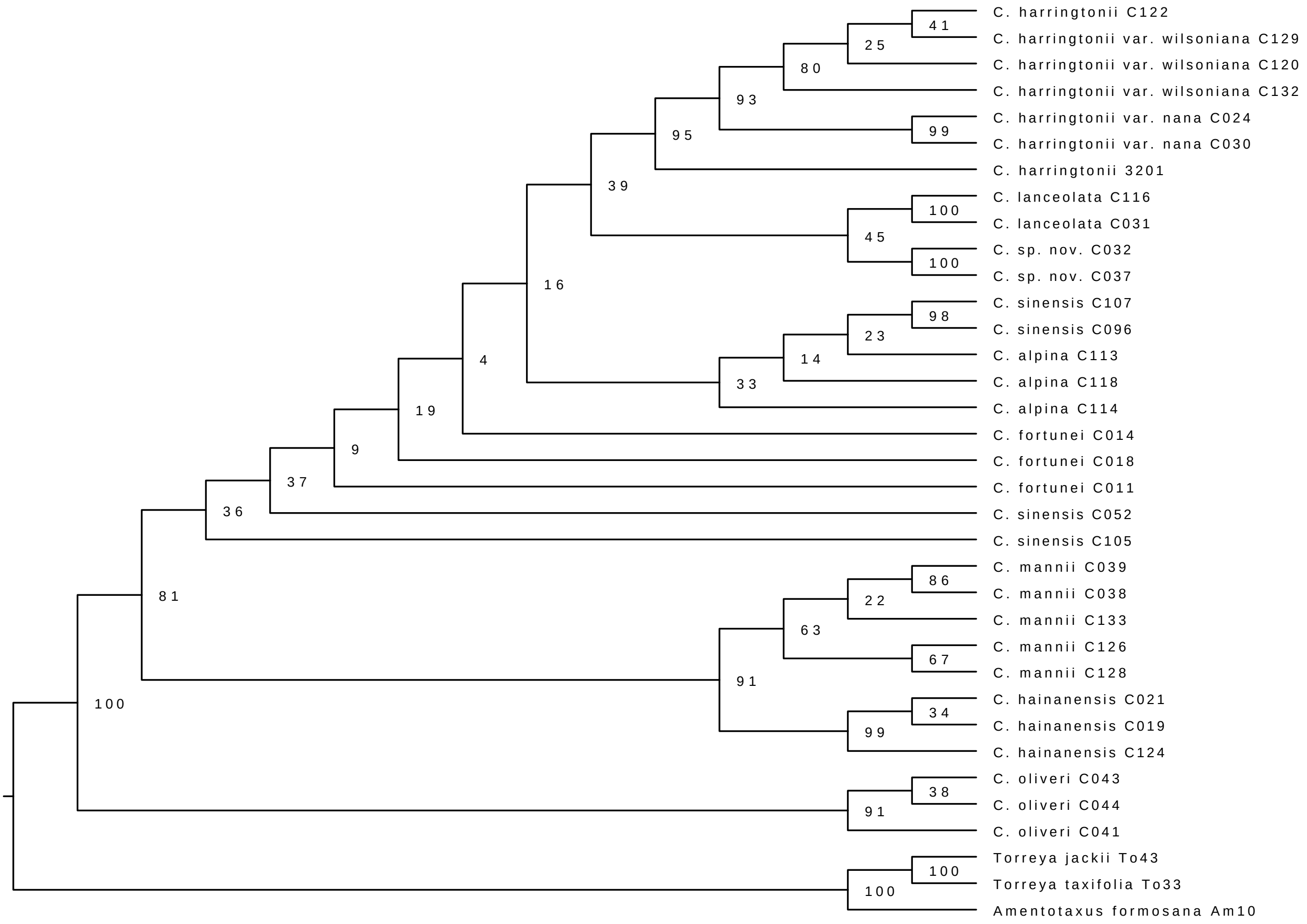

g: *ycf*2

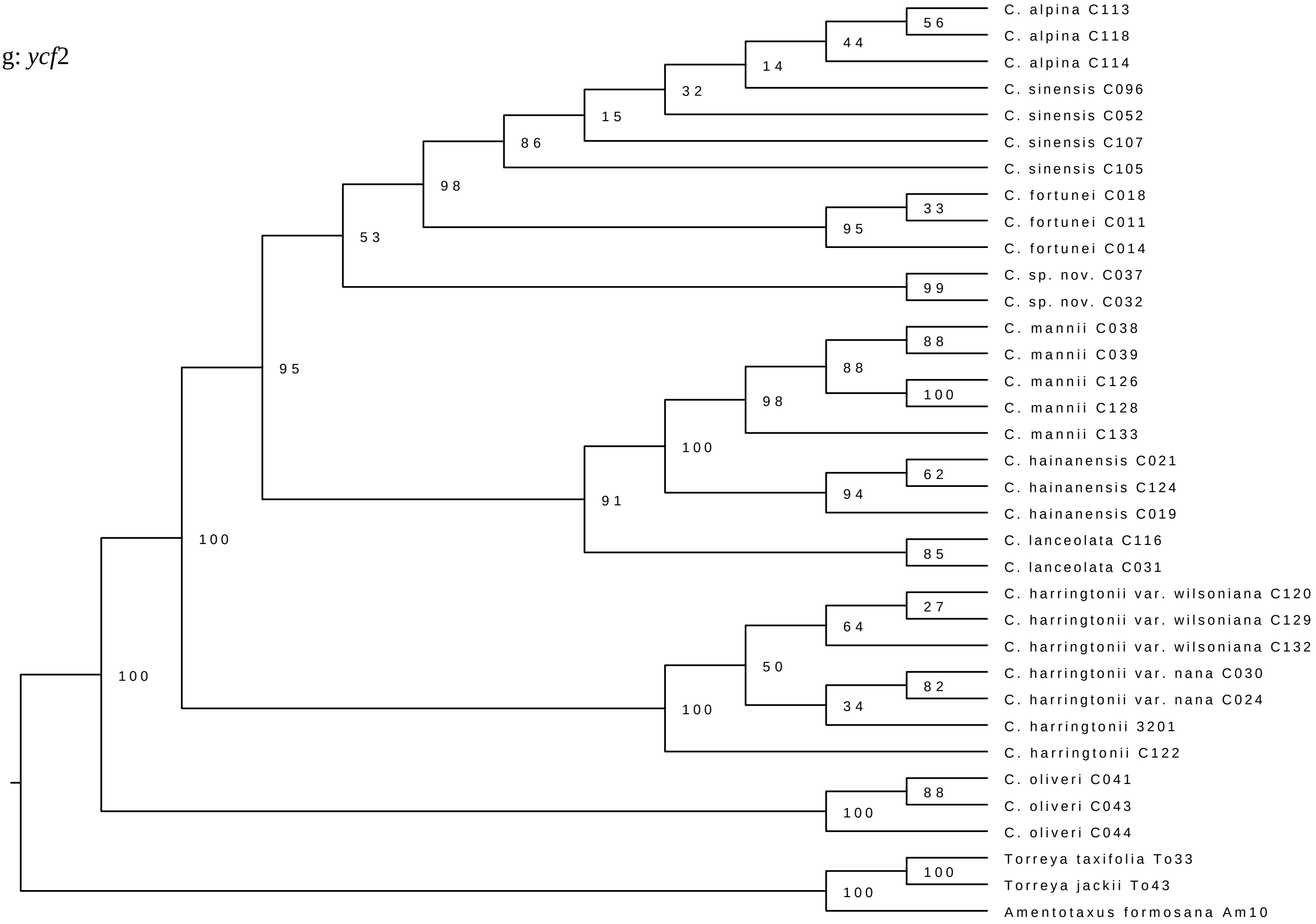

h: *ndh*F-*trn*R

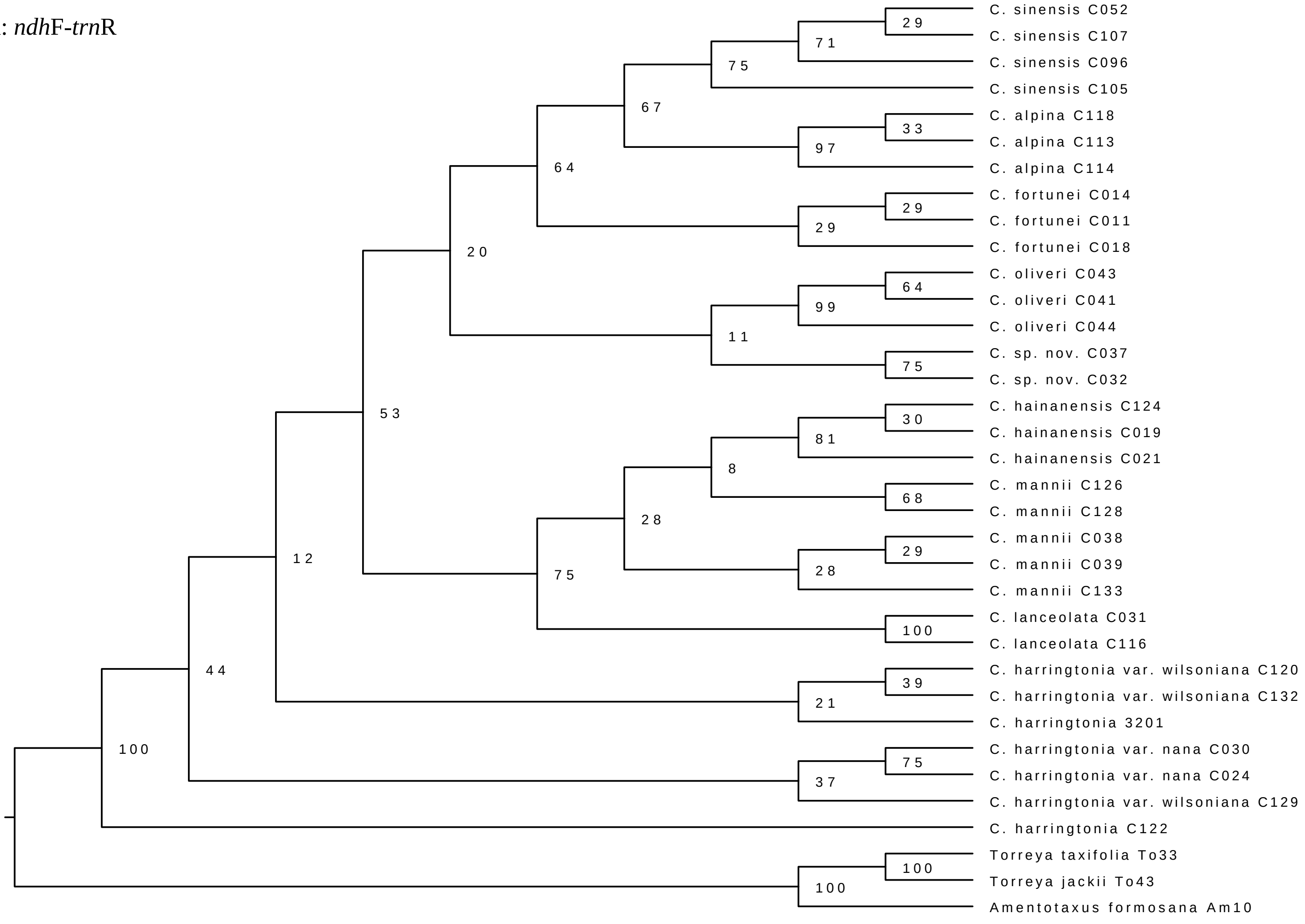

i: *mat*K + *rbc*L + *trn*H-*psb*A + *trn*L-*trn*F

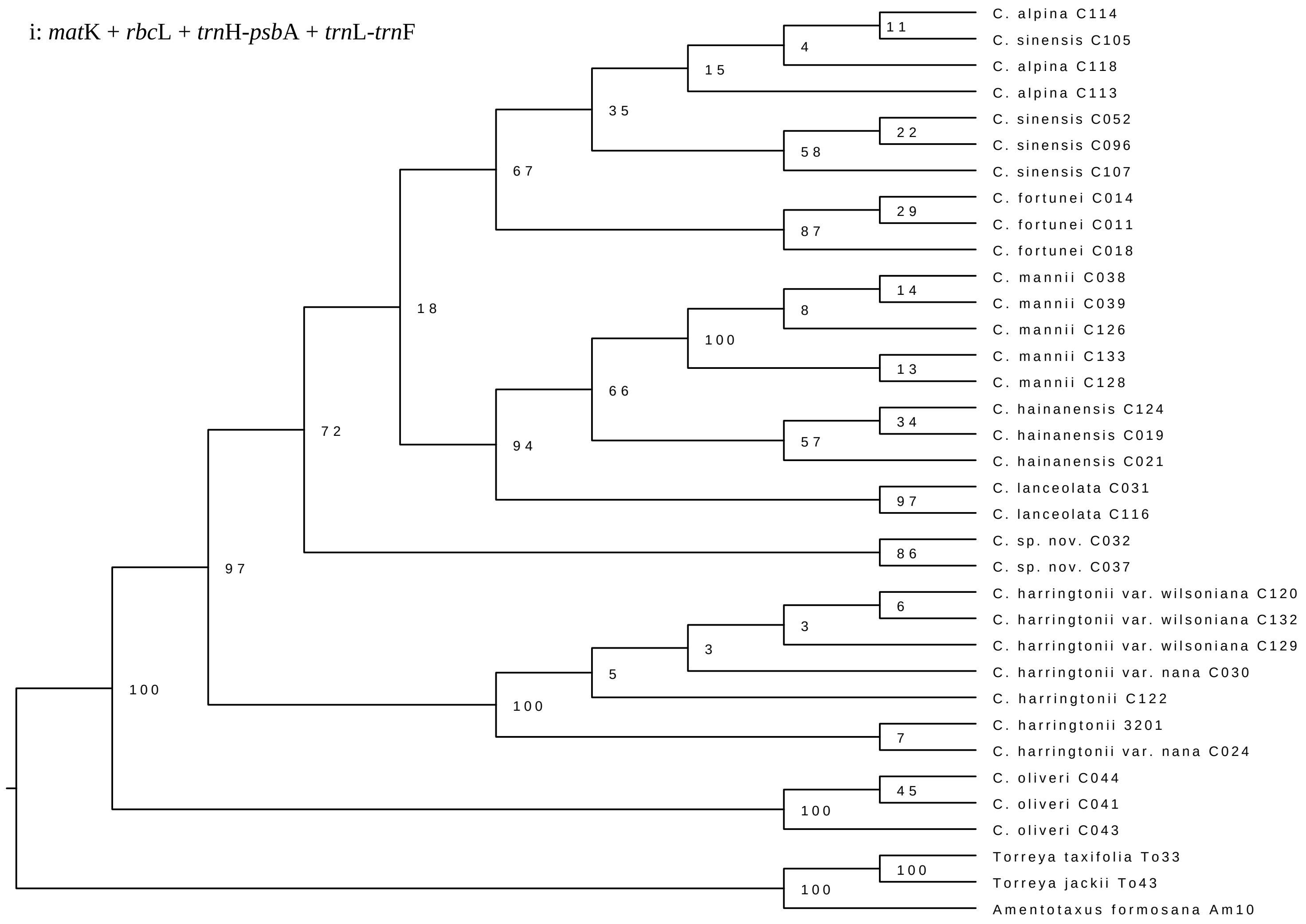

j: *mat*K

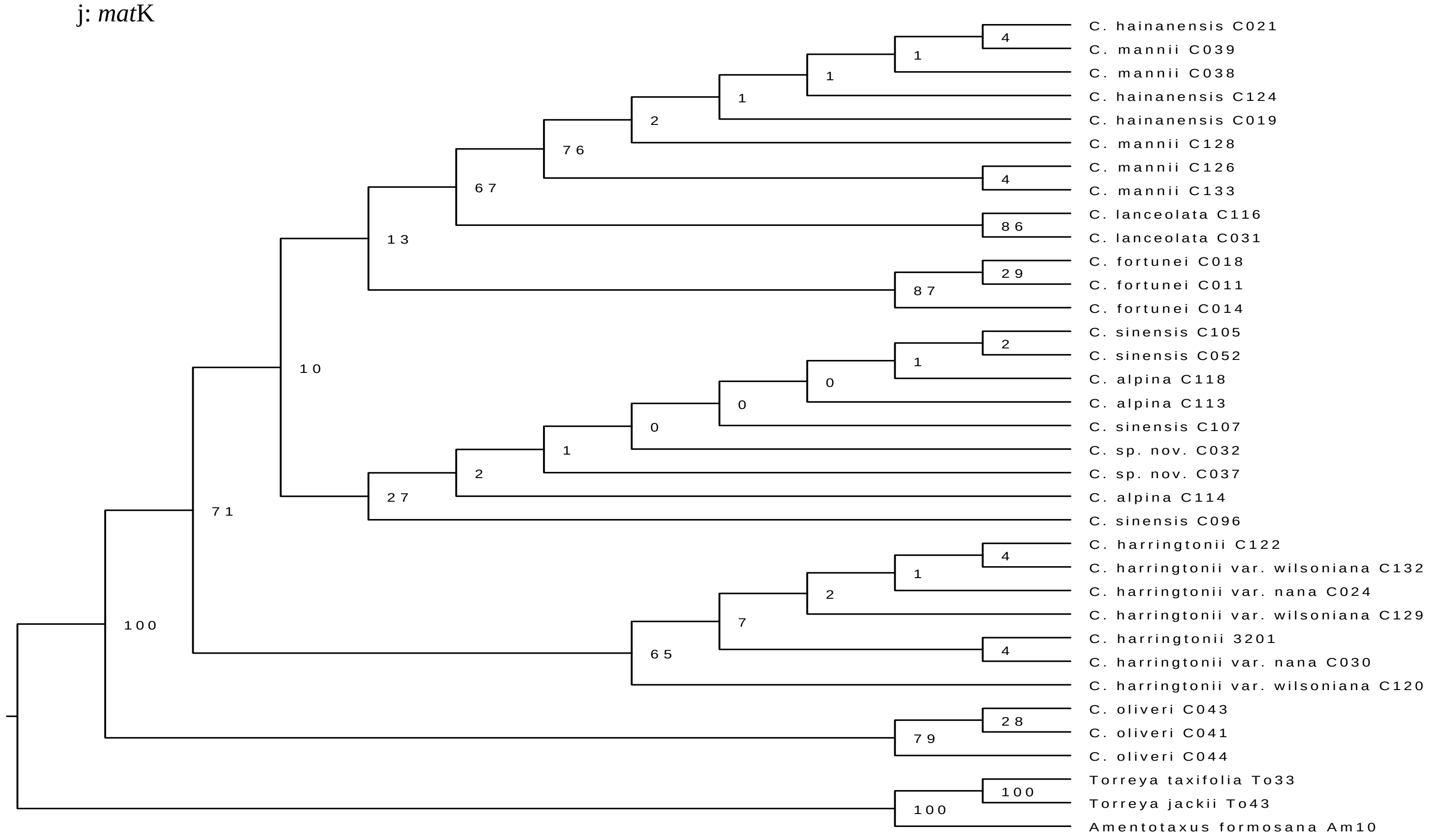

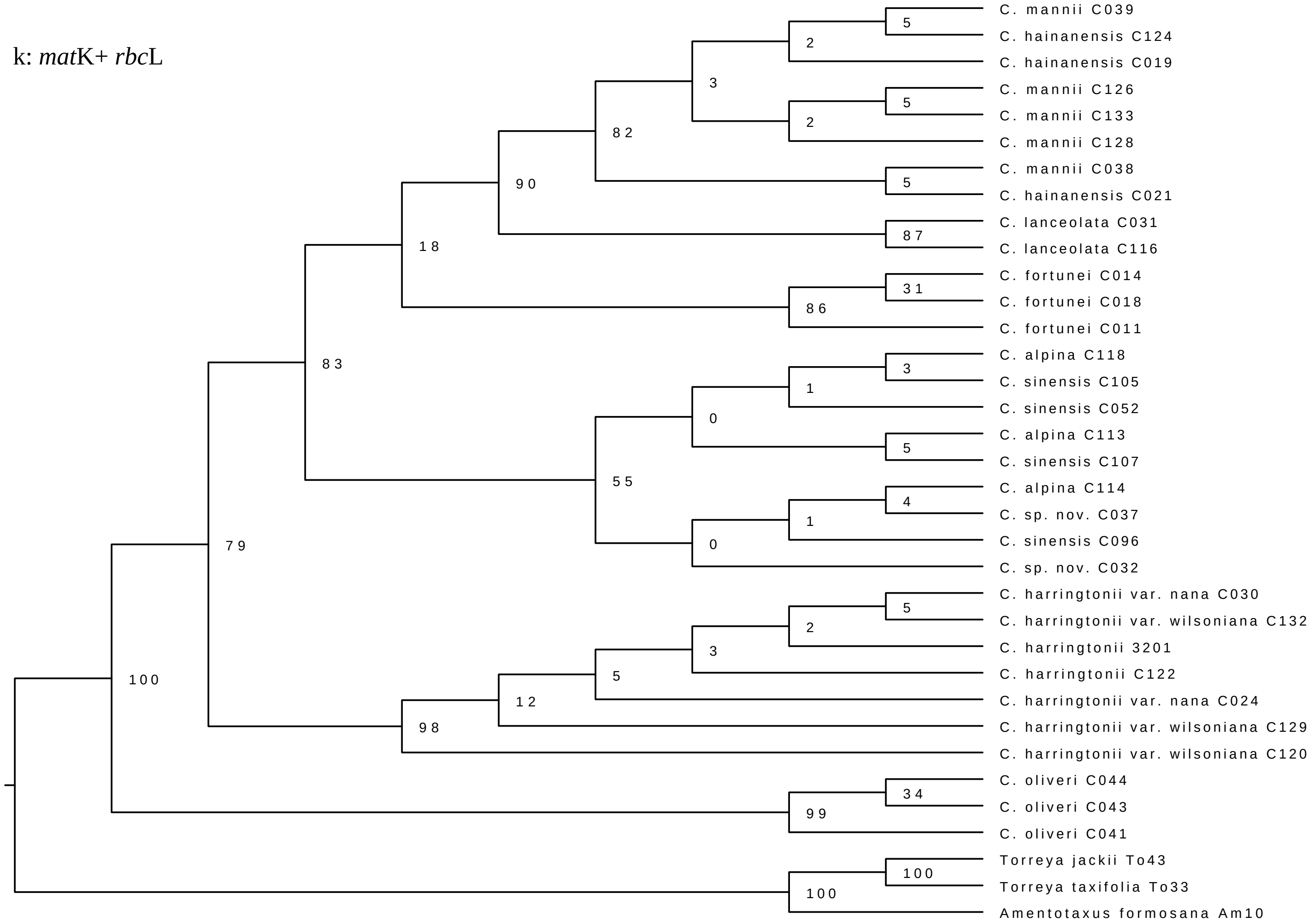
k: matK+ rbcL
C. mannii C039
C. hainanensis C124
C. hainanensis C019
C. mannii C126
C. mannii C133
C. mannii C128
C. mannii C038
C. hainanensis C021
C. lanceolata C031
C. lanceolata C116
C. fortunei C014
C. fortunei C018
C. fortunei C011
C. alpina C118
C. sinensis C105
C. sinensis C052
C. alpina C113
C. sinensis C107
C. alpina C114
C. sp. nov. C037
C. sinensis C096
C. sp. nov. C032
C. harringtonii var. nana C030
C. harringtonii var. wilsoniana C132
C. harringtonii 3201
C. harringtonii C122
C. harringtonii var. nana C024
C. harringtonii var. wilsoniana C129
C. harringtonii var. wilsoniana C120
C. oliveri C044
C. oliveri C043
C. oliveri C041
Torreya jackii To43
Torreya taxifolia To33
Amentotaxus formosana Am10

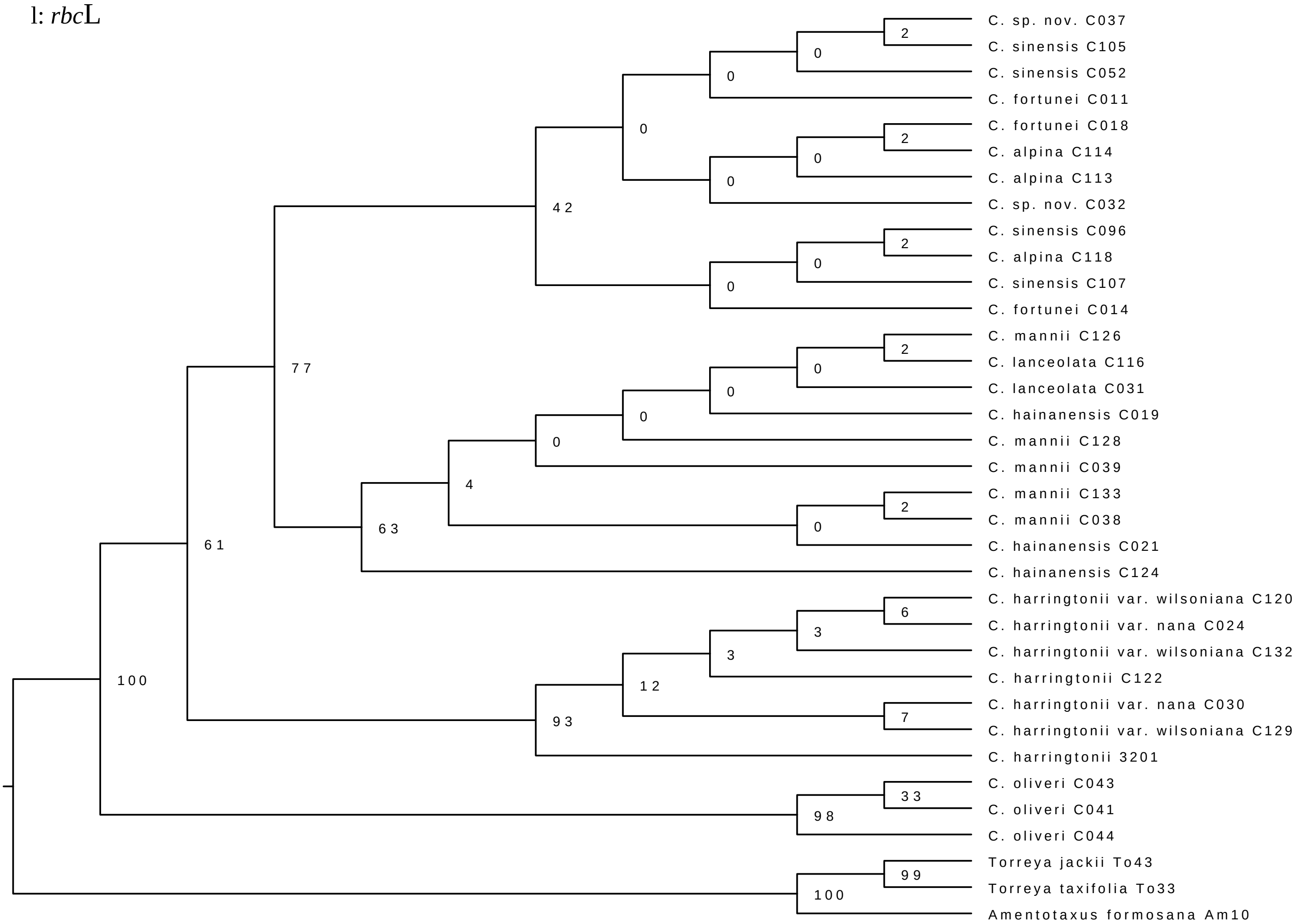
l: rbcL
C. sp. nov. C037
C. sinensis C105
C. sinensis C052
C. fortunei C011
C. fortunei C018
C. alpina C114
C. alpina C113
C. sp. nov. C032
C. sinensis C096
C. alpina C118
C. sinensis C107
C. fortunei C014
C. mannii C126
C. lanceolata C116
C. lanceolata C031
C. hainanensis C019
C. mannii C128
C. mannii C039
C. mannii C133
C. mannii C038
C. hainanensis C021
C. hainanensis C124
C. harringtonii var. wilsoniana C120
C. harringtonii var. nana C024
C. harringtonii var. wilsoniana C132
C. harringtonii C122
C. harringtonii var. nana C030
C. harringtonii var. wilsoniana C129
C. harringtonii 3201
C. oliveri C043
C. oliveri C041
C. oliveri C044
Torreya jackii To43
Torreya taxifolia To33
Amentotaxus formosana Am10

m: *trn*H-*psb*A

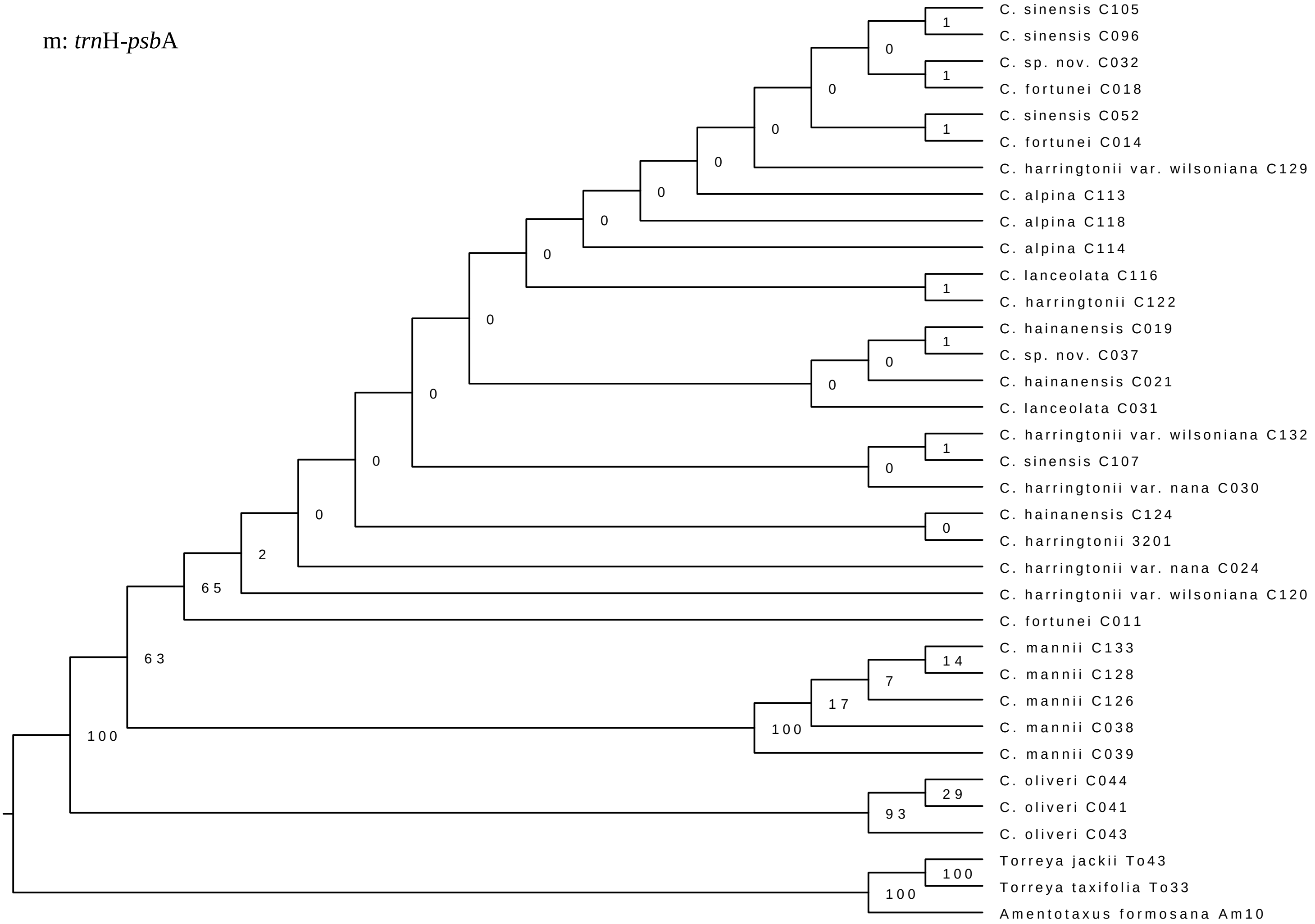

n: *trn*L-*trn*F

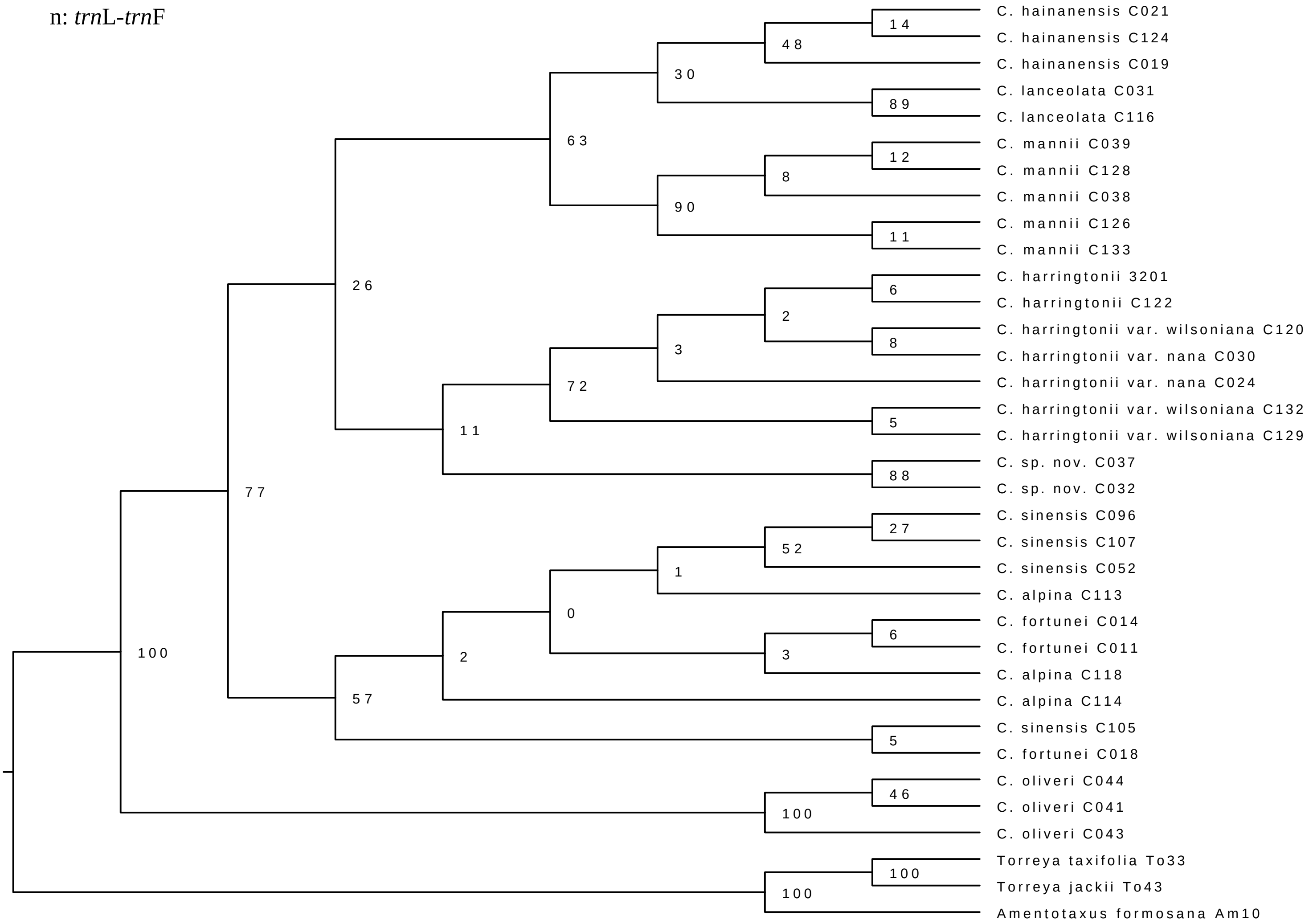

Supplement: Supplementary Figure S2 — Maximum Likelihood phylogenetic trees of 32 Cephalotaxus samples based on the eight highly variable plastid loci, the four single standard DNA barcode and two combinations of standard DNA barcodes (a. trnI-rrn16, b. ycf1, c. chlN-ycf1, d. clpP-accD, e. rps16, f. accD, g. ycf2, h. ndhF-trnR, i. matK + rbcL + trnH-psbA + trnL-trnF, j. matK, k. matK + rbcL, l. rbcL, m. trnH-psbA, n. trnL-trnF). Numbers to the right of nodes indicate bootstrap support values. [file Data_Sheet_2.PDF]
